# Acceptance of AI in Health Care for Short- and Long-Term Treatments: Pilot Development Study of an Integrated Theoretical Model

**DOI:** 10.2196/48600

**Published:** 2024-07-18

**Authors:** Johannes Wichmann, Tanja Sophie Gesk, Michael Leyer

**Affiliations:** 1 Working group Digitalization and Process Management Department of Business Philipps-University Marburg Marburg Germany; 2 Management Department Queensland University of Technology Brisbane Australia

**Keywords:** health information systems, integrated theoretical model, artificial intelligence, health care, technology acceptance, long-term treatments, short-term treatments, mobile phone

## Abstract

**Background:**

As digital technologies and especially artificial intelligence (AI) become increasingly important in health care, it is essential to determine whether and why potential users intend to use related health information systems (HIS). Several theories exist, but they focus mainly on aspects of health care or information systems, in addition to general psychological theories, and hence provide a small number of variables to explain future behavior. Thus, research that provides a larger number of variables by combining several theories from health care, information systems, and psychology is necessary.

**Objective:**

This study aims to investigate the intention to use new HIS for decisions concerning short- and long-term medical treatments using an integrated approach with several variables to explain future behavior.

**Methods:**

We developed an integrated theoretical model based on theories from health care, information systems, and psychology that allowed us to analyze the duality approach of adaptive and nonadaptive appraisals and their influence on the intention to use HIS. We applied the integrated theoretical model to the short-term treatment using AI-based HIS for surgery and the long-term treatment of diabetes tracking using survey data with structured equation modeling. To differentiate between certain levels of AI involvement, we used several scenarios that include treatments by physicians only, physicians with AI support, and AI only to understand how individuals perceive the influence of AI.

**Results:**

Our results showed that for short- and long-term treatments, the variables perceived threats, fear (disease), perceived efficacy, attitude (HIS), and perceived norms are important to consider when determining the intention to use AI-based HIS. Furthermore, the results revealed that perceived efficacy and attitude (HIS) are the most important variables to determine intention to use for all treatments and scenarios. In contrast, abilities (HIS) were important for short-term treatments only. For our 9 scenarios, adaptive and nonadaptive appraisals were both important to determine intention to use, depending on whether the treatment is known. Furthermore, we determined *R*² values that varied between 57.9% and 81.7% for our scenarios, which showed that the explanation power of our model is medium to good.

**Conclusions:**

We contribute to HIS literature by highlighting the importance of integrating disease- and technology-related factors and by providing an integrated theoretical model. As such, we show how adaptive and nonadaptive appraisals should be arranged to report on medical decisions in the future, especially in the short and long terms. Physicians and HIS developers can use our insights to identify promising rationale for HIS adoption concerning short- and long-term treatments and adapt and develop HIS accordingly. Specifically, HIS developers should ensure that future HIS act in terms of HIS functions, as our study shows that efficient HIS lead to a positive attitude toward the HIS and ultimately to a higher intention to use.

## Introduction

### Overview

New digital technologies offer the opportunity to provide more connected health services to individuals [1]. Health information systems (HIS) building on these digital technologies provide different health services to individuals. They can help to identify diseases and support individual treatment faster [2]. HIS exist in various forms with different levels of automation, as the share of HIS using artificial intelligence (AI) is rising. AI-based HIS are self-learning and improve their own algorithms on the basis of an ongoing evaluation of the data entered [3]. As such, and until now, AI-based HIS have mostly been used to support physicians in their decisions concerning medical treatments toward patients, for example, in radiology [4]. However, AI-based HIS can also be used by patients to monitor their long-term treatments, for example, concerning cancer detection and treatment [5]. Nonetheless, the use of AI-based HIS nowadays is still substantially lower than non–AI-based HIS [1]. Thus, while predicting the use of such future systems is important [6], investigating the use of AI-based HIS is of particular importance to maintaining successful HIS adoption in the future [7]. By doing so, AI-based HIS shall be able to adapt to new situations, which is especially useful and more efficient in health care applications [8].

In health care, there are 2 different perspectives that have to be balanced to explain intention and behavior and thus are relevant for investigating health-related perspectives. The term for these 2 perspectives is “duality approach” [9,10]. First, the disease is considered, and how individuals root their behavior in this regard is referred to as “adaptive appraisals.” Second, individuals evaluate the behavior toward the intervention in question to counter the disease, which is called “nonadaptive appraisals” [11]. Current research shows that the duality approach of balancing adaptive and nonadaptive appraisals is sufficient and important for AI-based HIS [3]. Investigating such appraisals in health care is of particular importance, as individuals often come into physical contact with information systems [12].

To investigate these 2 perspectives, we combined theories of different areas to have an interdisciplinary view. Namely, they are the Health Belief Model (HBM), the Protection Motivation Theory (PMT), the Extended Parallel Process Model (EPPM) from a health care perspective, the Unified Theory of Acceptance and Use of Technology 2 (UTAUT2) from a technological perspective, and the reasoned action approach (RAA) from a psychological perspective. By combining these theories, it becomes evident that the theories share variables for their explanation process, although they are arranged in different ways. Such combinations have already been used to develop integrated frameworks to predict the intention to use AI-based HIS, for example, by Gesk et al [13,14] and Chu and Liu [15]. Nonetheless, all the approaches only consider short- or long-term treatments while failing to compare and draw conclusions for both. This is important as one-point-in-time decisions differ from ongoing decisions that accompany individuals every day [16,17].

Thus, our research question to fill this gap is which factors are relevant for the intention to use AI-based HIS for (1) short-term treatments and (2) long-term treatments?

To answer this question, we design an integrated framework using the duality approach of adaptive and nonadaptive appraisals. We do so as we apply our framework to the diseases such as cataracts, arthrosis, and diabetes. All diseases are non–life-threatening but limit the quality of life [18-20]. They, however, differ in terms of standard procedures. Arthrosis operations typically follow less-standardized protocols as fewer people are affected [13,19,21], cataract surgeries are conducted with standard procedures for a large number of individuals [13,22,23], and diabetes follows a continuous treatment approach for which the options are also quite standardized. Hence, cataracts and arthrosis are considered short term; for example, surgeries that are supported by AI can cure these diseases for at least a certain amount of time [19,22]. In contrast, diabetes is considered long term; for example, treatment plans that are supported by AI can at least reduce complaints that are caused by diabetes [24].

To investigate the intention to use AI-based HIS for short- and long-term treatments, we surveyed 693 individuals in total, who were assigned to the 3 diseases and 3 related AI-based HIS scenarios. First, physicians perform the medical treatment on their own. Second, physicians are supported by AI. Third, AI performs medical treatment on its own. Our results showed that with the increasing support of AI, adaptive appraisals become more important than nonadaptive appraisals for both short- and long-term treatments. Thus, individuals do not consider the disease in balancing their appraisals anymore; instead, they evaluate the treatment based on information concerning the AI-based HIS. Hence, this study provides important implications for business and research as we give insights into how information for AI-based HIS should be distributed.

This study is organized as follows: first, we define HIS. Second, we provide an overview of relevant theories in health care, information systems, and psychology and combine the relevant elements to design our integrated theoretical model. Third, we present materials and methods, in which we apply our integrated model to short- and long-term treatments with different scenarios. We then report the results of our study, which are then discussed concerning short- and long-term treatments and our integrated theoretical model. This study concludes with theoretical and practical implications and limitations, as well as directions for future work.

### Theoretical Background

#### AI-Based HIS

HIS are dedicated to assisting in health care services that assist physicians and patients in short- and long-term medical treatments [25]. While HIS are relevant for medical treatments for decades now, AI-based HIS are one of the most promising developments of HIS in recent years [3,8]. HIS are AI based when they require machine learning effects in performing their main task, such as problem-solving, shot-following, perception, and communication [26]. Thus, AI-based HIS differ from other HIS in that the AI-based HIS are able to perform self-learning processes [3]. Hence, AI-based HIS aim to identify patterns and to derive implications from valid, novel, and valuable data sets, whereas machine learning algorithms aim to continuously improve those patterns [5,27].

For surgeries in arthrosis [28] and cataracts [22] and short-term treatments, AI-based HIS that support physicians in their performance exist. They aim to identify patterns better than physicians to improve those surgeries in the future [22,28,29]. Concerning long-term treatments and diabetes, AI-based HIS to support physicians and patients exist, for example, by defining individual intervention plans considering diabetes issues [20,24]. Furthermore, existing studies addressing AI-based HIS cover aspects of the theories we used in our integrated model. For instance, Palmisciano et al [30] examined the attitudes of patients and their families toward the use of AI in neurosurgery as a short-term treatment. As a result, most patients assume health care benefits when AI is used as an assistant in neurosurgery. Furthermore, Tran et al [31] investigated patients’ views concerning the use of wearable devices that use AI-based HIS for long-term treatments. They determined that only a minority of their patients would accept getting treated by an AI-based HIS that is not surveilled by a physician. As another example of long-term treatments, Broadbent et al [32] and Dziergwa et al [12] investigated the acceptance of social robots for the care of older adults. According to Broadbent et al [32], the acceptance of these robots tends to be lower among older adults; however, according to Dziergwa et al [12], age is less relevant regarding acceptance. Moreover, Longoni et al [1] further investigated the reasons for patients’ rejection of the use of AI in health care. They determined that the most important reason for rejecting the use of AI is perceived personal uniqueness in relation to each individual’s medical history. In doing so, individuals believe that AI is unable to determine the most appropriate treatment for an individual with a nonaverage medical history [1].

#### Related Work

Research on AI-based HIS for short- and long-term medical treatments exists and is of rising interest [7]. For our study, we used diseases that are already AI supported to improve comprehensibility and that are not life-threatening. As the first disease, we considered “arthrosis,” which is a degenerative joint disease that causes pain in the foot. While an intervention (eg, surgery) is not necessary, it is still recommended to be able to walk pain free [19]. Concerning the second disease, we considered “cataract,” which slowly deteriorates the eye vision of an individual, and it is one of the leading causes of visual impairment worldwide [22]. Surgery can be performed to counter the disease and to significantly improve eye vision [22]. As the third disease, we considered “diabetes type 1,” which is a lifelong disease that requires individuals to track and add the hormone insulin to their body to digest appropriately [20]. Mandatory for tracking insulin is the blood sugar level, which can be better observed by using AI-based HIS [20]. For our investigation, we used these diseases to design scenarios. As such, we take on an intervention perspective concerning AI, as we consider the common separation of AI in augmentation and automation [33]. We do so as we divided our 3 diseases into different scenarios, in which the treatment is performed by (1) physicians only, (2) physicians supported by AI, and (3) AI only, as recommended by Ward [34]. Furthermore, Longoni et al [1] stated that research that addresses new health technologies should use different scenarios, as they investigated the trustworthiness of physician- and AI-based medical treatments. Their results showed that individuals are more likely to rely on recommendations from physicians than from AI, assuming that physicians perform better in evaluating an individual’s medical history. Approaches to automated treatments are not new; for example, Palmisciano et al [30] investigated the attitudes of patients and their families toward AI in neurosurgery. They found that patients expressed concerns about autonomous surgical interventions. Patients indicated that maintaining human interaction during medical treatments was very important to them and that they wanted humans to monitor AI. Consequently, the individual’s perception of the effectiveness of the AI-based intervention is an important variable in predicting the intention to use. These propositions were supported by Esmaeilzadeh [7], who investigated the use of various AI-based tools for health care purposes via a survey study using patients’ perspectives. To determine whether people favor AI over physicians in medicine concerning radiology, robotic surgery, and dermatology, Yakar et al [35] conducted a survey. They also determined that a general attitude toward AI is important for intention to use, as is distrust and accountability, personal interaction, and efficiency, with younger individuals having a higher intention to use. While Yakar et al [35] determined similar values for radiology, surgery, and dermatology, their main finding was that distrust toward AI performance is the main driver for people favoring physicians over AI in medicine. Next to behavioral evidence using surveys, Yun et al [36] also used neural evidence on consumer responses to human physicians and medical AI. They determined that emotions play a crucial role in humans deciding whether they follow the recommendations of human physicians or AI to perform medical treatments. People are more likely to follow the recommendations of AI if their primary diagnosis and medical statements are conducted in a personalized, emphasized way instead of a mechanical conversation. In contrast to recommendations issued by human physicians, it did not matter whether primary diagnoses and medical statements were personalized or mechanical, as the likelihood was similar. Furthermore, to generate positive emotions toward HIS treatments and to reduce pain, Ahmadpour et al [37] recommended the use of augmented reality. As such, patients should be distracted using augmented reality videos of beaches and nature scenes [38] that ease them and have proven to be effective during medical treatments. To investigate the requirements and expectations of physicians in German university hospitals toward future medical AI applications, Maassen et al [39] conducted a web-based survey. They determined that their participants were technically affinitive, and the more affinitive they are, the better their overall rating of AI is. In general, physicians perceive that AI will be beneficial in medication and therapy as well as imaging procedures, while AI will not be that beneficial for diagnostic purposes. According to them, the importance of physician-AI corporations in the future will rise, and they wish for opportunities to evaluate their patient data by AI on an anonymous basis. In contrast to the study by Maassen et al [39] investigating German physicians, Fritsch et al [40] surveyed German patients and their attitudes and perceptions of AI in health care. While their previous knowledge of AI was limited, the patients felt positive about the use of AI in medicine. Nonetheless and in accordance with other studies [30,36], attitude is important for the intention to use in German society [35]. By investigating the use of wearable devices in health care, Gao et al [18] underpin the importance of perceived effectiveness in AI-based HIS research. They found that most patients are familiar with the use of variable devices (such as a smartphone app). In addition, they determined that the intention to use such wearable devices is dependent on perceived threats and perceived effectiveness toward the AI-based HIS. Furthermore, for long-term treatments, Broadbent et al [32] determined that the acceptance of social care robots by older adults is low, as they are rather hesitant toward new technologies. In contrast, Dziergwa et al [12] found that due to positive attitudes toward AI-based HIS, the acceptance of social care robots by older adults is high. While the study of Broadbent et al [32] was conducted in 2009, the study by Dziergwa et al [12] was published in 2017, which also displays changes in the overall perception of AI-based HIS in the recent past. While the overall attitude toward AI-based HIS improved in society over the last years, training toward the use of such systems also improves the overall perception, as determined by Sit et al [5].

#### Combining Theories From Health Care, Information Systems, and Psychology

As we aimed to explain psychological reasons for patients to use AI-based HIS for short- and long-term treatments, we had to combine theories from those areas. We thus focused on theories that predict the intention to use, as the intention is known to be the most important predictor for use behavior [41]. Thus, we integrated the following theories into our integrated model: first, from the health care perspective, we used the HBM that was developed by Hochbaum [42] and Rosenstock [43,44]. The HBM seeks to understand the adoption of health care measures. The model focuses on perceived threats and behavioral evaluations of preventive actions as the 2 drivers of intention to engage in health-related behavior [45].

Second, we used the PMT, which is a further development of the HBM and was conceptualized by Rogers [9] and Floyd et al [46]. The PMT states that intention is mainly influenced by adaptive and nonadaptive appraisals, called the duality approach. According to this duality approach, individuals balance between the treatment (as adaptive appraisals) and the disease (as nonadaptive appraisals) when determining their intention toward a certain behavior. Third, we used the EPPM, which also uses the duality approach and is similar to the PMT but uses a different behavioral reasoning [10,47]. In addition, the EPPM states that adaptive appraisals are reasoned to be more cognitive, whereas nonadaptive appraisals are more emotional [10,48]. Fourth, concerning theories to investigate information systems, we applied the UTAUT2 to our model. The UTAUT2 was developed by Venkatesh et al [49], who further developed the Technology Acceptance Model (TAM) in its various versions [49-53]. Using the TAM and UTAUT2 for investigations concerning information systems is very important, as they were sufficiently used to determine the intention to use information systems in the past [54].

Fifth, from a general psychological perspective, the Theory of Planned Behavior (TPB) is famous for investigating the intention toward a certain behavior [55]. It is similar to the Theory of Reasoned Action, which is why both theories were combined in the RAA. The RAA states that the intention toward a behavior is dependent on attitude, perceived norms, and perceived behavioral control, which, in turn, is dependent on associated beliefs [41].

Furthermore, theories from health care, information systems, and psychology have been combined in the past to determine the intention to use. For instance, Chau and Hu [50] used several theories to investigate physicians’ intention to use telemedicine applications, such as the TAM and TPB. Patients were investigated by Ku and Hsieh [17], who combined the TPB and HBM to understand patients’ intention to use health management mobile services. To investigate the intention to adopt mobile health services, Zhang et al [56] combined the TPB and PMT. Furthermore, the use of wearable technologies in health care was investigated by Gao et al [18], who combined the UTAUT2 with the PMT. However, research on an integrated model to investigate and confront AI-based HIS concerning short- and long-term treatments is still lacking.

#### Integrated Theoretical Framework

In our integrated theoretical framework, we combined different theories from health care, psychology, and information systems. As most of the theories use similar variables to determine a behavioral intention, Table 1 summarizes all variables used in our model and synonyms concerning those variables.

**Table 1:** Overview of variables in relevant theories.

Variables of integrated theoretical model (references) or synonym, theory, and references; definition in the integrated model	HBM^a^	PMT^b^	EPPM^c^	UTAUT2^d^	RAA^e^
*Nonadaptive appraisals* (fear control process [EPPM] [10]); reflect the individual’s evaluation of the disease. Nonadaptive rewards are based on threats that are composed of perceived severity and perceived susceptibility and influence fear, nonadaptive rewards for maintaining the current behavior toward the disease, as well as an individual’s attitude toward the disease.			✓		
Perceived severity [9,57]; refers to the likelihood an individual perceives coming down with the disease	✓	✓			
Perceived vulnerability [57] (probability of occurrence [PMT] [9]); describes the susceptibility an individual perceives toward coming with the disease	✓	✓			
Nonadaptive rewards [46] (maladaptive response rewards [PMT] [46]); reflect the benefits an individual receives by maintaining the current behavior toward the disease		✓			
Fear (disease) [9,46] (perceived threats, leading to fear [HBM] [57]); measures the emotion “anxiety” an individual feels toward the disease	✓	✓	✓		
Attitude (disease) [41,58]; describes the stance an individual has toward the disease					✓
*Adaptive appraisals* [9,10] (danger control process [EPPM] [10]); reflect the individual’s evaluation of the HIS^f^. Adaptive appraisals are based on the efficacy an individual perceives toward the HIS. In turn, this efficacy is composed of perceived HIS efficacy and perceived self-efficacy, which influence fear and attitude toward the HIS. Furthermore, perceived norms influence the adaptive appraisals of individuals.		✓	✓		
Perceived HIS efficacy [9,10] (perceived benefits [HBM] [44] or response efficacy [PMT] [9], [EPPM] [10], effort, performance expectancy, perceived usefulness, or hedonic motivation [49]); refers to the effectiveness of the HIS and the benefits provided to the individual	✓	✓	✓		
Perceived self-efficacy [9,10] (facilitating conditions [UTATU2] [49] or perceived behavioral control [RAA] [41]); describes the degree of freedom an individual recognizes while determining the intention to use an HIS		✓	✓	✓	✓
Perceived norms [41] (social forces [HBM] [42], social pressure [HBM] [43], advice from others [HBM] [57], verbal persuasion [PMT] [46], or social influence [UTAUT2] [49]); represent the opinions of others toward the HIS	✓	✓		✓	✓
Fear (HIS) [9,58] (anxiety [UTAUT2] [49]); explains the emotion “fear/anxiety” an individual feels toward the HIS		✓		✓	
Attitude (HIS) [41,49,58]; reflects an individual’s stance toward the HIS				✓	✓
Intention to use [10,41,49,58] (likelihood of taking action [HBM] [57], cues to action [HBM] [42,44], or protection motivation [PMT] [46]); refers to an individual’s preference toward using the HIS	✓	✓	✓	✓	✓

^a^HBM: Health Belief Model.

^b^PMT: Protection Motivation Theory.

^c^EPPM: Extended Parallel Processing Model.

^d^UTAUT2: Unified Theory of Acceptance and Use of Technology 2.

^e^RAA: reasoned action approach.

^f^HIS: health information systems.

**Figure 1:**
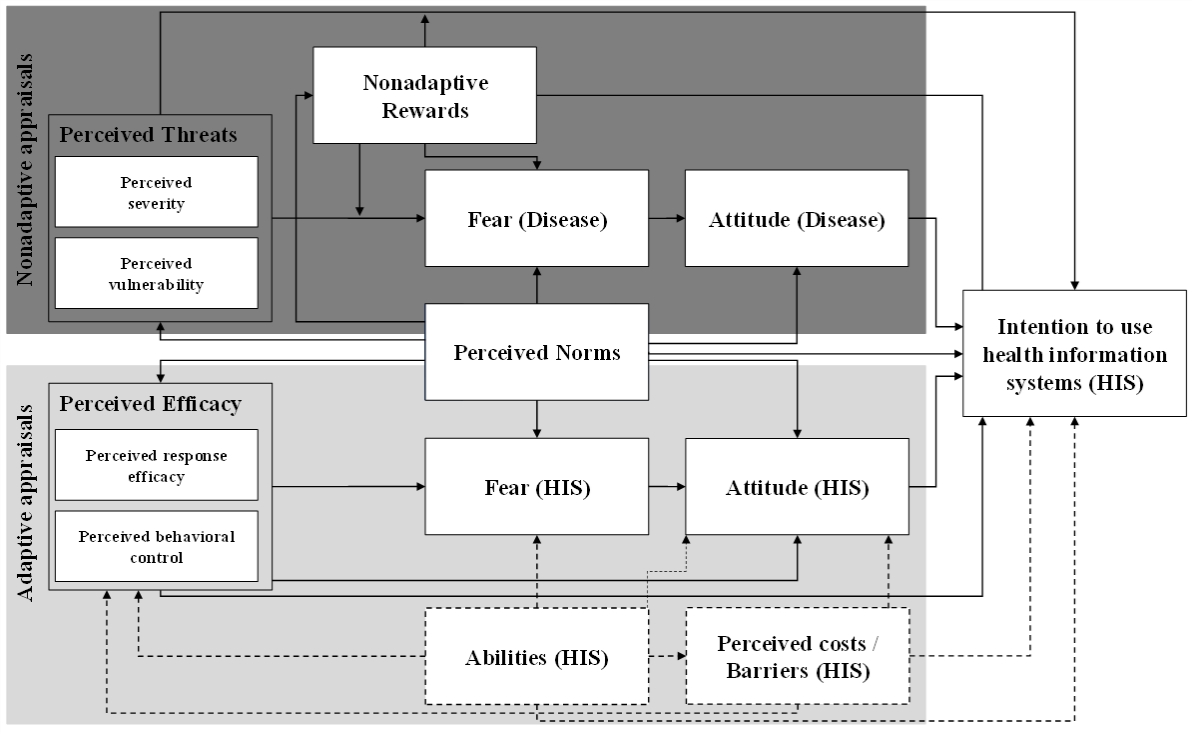
Integrated theoretical model.

Table 1 shows that the theories, models, and approaches share common elements that are conceptualized slightly differently. For our integrated model, we used the duality approach of Witte [10]. Thus, our model states that the intention to use is dependent on 2 paths of explanation, namely adaptive and nonadaptive appraisals. Hence, on the one hand, adaptive appraisals explain how individuals evaluate the treatment in question, that is, the AI-based HIS in our case. On the other hand, nonadaptive appraisals reflect how individuals assess the disease in question and potential threats relating to it. Balancing those appraisals is important if individuals tend to stick to a nonadaptive behavior. They do so if the benefits they receive by maintaining the current behavior (called nonadaptive rewards) outweigh the threats those individuals perceive toward the disease [9,43,44]. In turn, the perceived threats are composed of perceived severity and perceived vulnerability. Thus, perceived threats reflect the probability individuals assume toward coming down with the disease and, if so, how much the disease will impact them. Accordingly, perceived threats and nonadaptive rewards influence fear [58] toward the disease, which in turn influences attitude [41] toward the disease. Ultimately, all these variables influence the intention toward using the AI-based HIS from a disease-related perspective [9,41,43,44,58]. In contrast, concerning adaptive appraisals, the perceived efficacy of the AI-based HIS is an important root cause for the intention to use [49]. In turn, perceived efficacy is composed of perceived response efficacy and perceived behavioral control. Perceived response efficacy refers to the AI-based HIS itself in that individuals have to assess how sufficiently the AI-based HIS works to counter the disease [7,9]. Concerning perceived behavioral control, individuals have to ascertain if and how they actually influence their behavior in question, that is, if they are able to freely decide whether they intend to use the AI-based HIS [13,41]. Then, according to the duality approach [46], fear [58] and attitude [41] are important variables for adaptive appraisals. Hence, these variables, namely perceived efficacy, fear, and attitude, influence the intention to use AI-based HIS concerning adaptive appraisals. Furthermore, perceived norms concerning the treatment in question are important and influence both adaptive and nonadaptive appraisals. As such, perceived norms reflect the opinions of others toward the behavior in question, that is, how important peers of individuals would assess that individuals use AI-based HIS [41]. For a better understanding, Figure 1 presents our integrated model.

#### Hypotheses and Research Model

We apply the integrated theoretical model to our scenarios to provide empirical support for understanding the reasons behind the intention to use AI-based HIS. Therefore, we derive specific hypotheses that are enriched by empirical research at the application level. First, the specific disease and associated threat appraisals toward the specific disease are reflected in an individual’s perceived health threats due to a disease [9]. These threat appraisals influence the fear an individual feels about the disease [59]. Fear is an emotion that triggers anxious or emotional responses when a specific fear-inducing behavior is performed [49,60]. Therefore, we hypothesize that individuals at risk of the disease will (1) feel the emotion of fear more strongly [59] and (2) have a higher intention to use AI-based HIS for treatment to minimize or avert further negative health outcomes [46,59]. Consequently, our first hypothesis is as follows:

- Hypothesis 1: perceived threats have (1) a positive influence on fear (disease) and (2) a positive influence on intention to use.

As positive outcomes for refraining from the treatment in question, nonadaptive rewards may influence fear, as more positive outcomes lower fear toward the disease [61]. In addition, nonadaptive rewards reinforce nonadaptive behavior by preventing the use of AI-based HIS [46]. Hence, nonadaptive rewards may lower the intention to use AI-based HIS [61]. Consequently, our second hypothesis is as follows:

- Hypothesis 2: nonadaptive rewards have a negative influence on (1) fear (disease) as well as (2) intention to use.

Furthermore, attitudes are opinions that are positively or negatively associated with a particular disease [49]. Fear as an emotion is known to lower attitudes toward the disease [46]. Thus, we hypothesize that attitudes (disease) will be negatively influenced by the emotion of fear, leading to the third hypothesis:

- Hypothesis 3: fear (disease) has a positive influence on attitude (disease).

As attitudes toward the disease are one of the most important drivers of intention to use [41], negative attitudes toward the disease will lead to a higher intention to use AI-based HIS. Therefore, our fourth hypothesis is as follows:

- Hypothesis 4: attitude (disease) has a positive influence on intention to use.

Second, we consider adaptive appraisals and focus on our treatments, that is, physicians only, physicians supported by AI, and AI only. In the beginning, perceived efficacy in relation to the intervention describes an individual’s perception that the treatment is useful to address a specific disease [49]. Perceived efficacy is one of the key elements related to the intention to use new technologies in health care [62]. In addition, perceived efficacy is defined by a high level of perceived behavior control and may lead to a higher willingness to use an intervention [18,62,63]. Treatments, such as surgeries performed by AI, are more efficient, more accurate, and less error-prone than humans as surgeons in most cases [8,64]. Following the duality approach of our integrated theoretical model, we also consider fear (HIS) and attitude (HIS) as variables of intention to use AI-based HIS. Therefore, our fifth hypothesis is as follows:

- Hypothesis 5: perceived efficacy has a negative influence on (1) fear (HIS) as well as a positive influence on (2) attitude (HIS) and (3) intention to use.

In studies of technology acceptance, fear (HIS) has a large impact on attitudes (HIS) toward technology and, ultimately, intentions to use it [30,64]. Furthermore, if individuals perceive anxiety, their willingness to accept AI for medical treatments is lower [60].

Therefore, we formulated the sixth hypothesis:

- Hypothesis 6: fear (HIS) has a negative influence on attitude (HIS).

Ultimately, attitude (HIS) has a significant role in technology acceptance in relation to the intention to use [30,41,53]. This leads to the following seventh hypothesis:

- Hypothesis 7: attitude (HIS) has a positive influence on intention to use.

In addition to the duality approach, we assume that social pressure regarding a certain behavior is defined as perceived norms [18,65]. Perceived norms influence all variables in the integrated theoretical model. Previous studies have already shown a positive influence of perceived norms on the intention to use new technologies in health care [62,66]. Therefore, we assume that adaptive appraisals related to AI-based HIS will be positively influenced. Furthermore, we expected that perceived norms would also make nonadaptive appraisals less attractive. Hence, our eighth hypothesis is as follows:

- Hypothesis 8: perceived norms have, on the one side, a positive influence on (1) perceived threats, (2) fear (disease), (3) intention to use, (4) attitude (HIS), and (5) perceived efficacy and, on the other side, a negative influence on (6) attitude (disease), (7) fear (HIS), and (8) maladaptive rewards.

According to the RAA by Fishbein and Ajzen [41], abilities are important for determining the intention to use HIS. As such, high abilities in our case reflect that individuals understand the specifications and risks of AI-based treatments quickly, which increases intention to use [7]. Hence, our ninth hypothesis is as follows:

- Hypothesis 9: abilities toward HIS positively influence (1) perceived efficacy, (2) fear (HIS), (3) attitude (HIS), (4) perceived costs or barriers, and (5) intention.

Furthermore, Venkatesh et al [49] stated that cost or barriers toward information systems are important variables to consider for evaluating the intention to use HIS. If individuals perceive high costs or barriers toward the treatment, their intention to use is lower [7]. Thus, our 10th hypothesis is as follows:

- Hypothesis 10: cost or barriers toward HIS negatively influence (1) perceived efficacy, (2) attitude (HIS), and (3) intention.

## Methods

### Measures

For our study, we used 3 different scenarios: (S1) arthrosis, (S2) cataracts, and (S3) diabetes type 1. Each scenario investigates different treatments that are performed (1) by physicians, (2) by physicians with AI support, and (3) by AI only. We used quantitative surveys for our study. First, we introduced the respective scenario using explanations shown in Multimedia Appendix 1. To ensure that our questions were answered attentively and correctly, we used attention checks according to Goodman et al [67]. Specifically, participants had to pass initial test questions to continue. We aligned the questionnaire according to the variables in the integrated model. The reflective variables are intention to use (3 items adapted from Fishbein and Ajzen [41]), attitude (HIS and disease; 5 items each adapted from Fishbein and Ajzen [41]), perceived norms (4 items adapted from Fishbein and Ajzen [41]), perceived behavioral control (4 items adapted from Fishbein and Ajzen [41]), perceived response efficacy (3 items adapted from Taheri-Kharameh et al [68]) as well as fear (HIS and disease; 3 items each adapted from Izard et al [69]), and nonadaptive rewards (3 items adapted from Vance et al [61]). The formative variables are perceived vulnerability (4 items adapted from Ku and Hsieh [17]) and perceived severity (7 items adapted from Ku and Hsieh [17]). All items were measured on a 7-point Likert scale. In addition, experience with AI-based services, general computer skills, and technical knowledge about AI are self-developed single-item control variables, which were measured with a 5-point Likert scale next to basic demographics. The complete questionnaire can be found in Multimedia Appendix 1.

### Participants and Data Collection

For gathering empirical data, we used the crowdworking platform Clickworker, which is similar to Amazon MTurk. On the one side, we asked 496 probands hypothetically having (S1) arthrosis and (S2) cataracts. We used this approach as we aimed to investigate medical decisions that are short term, that is, individuals had to decide whether they intend to use AI or physicians for treatment. Furthermore, they should be able to comply with nonadaptive appraisals, that is, to decide to have no treatment, which is possible for arthrosis and cataracts [19,22]. For S1 and S2, a total of 40.7% (203/498) of the participants were female, 59.1% (294/496) were male, and 0.2% (1/496) did not specify their sex. On the other side, 197 probands actually had type 1 diabetes (S3). Hence, we took a different approach to long-term treatments, as we had to ensure that individuals actually have the disease to be able to judge differences concerning medical treatments performed by AI or physicians. Hence, we investigated participants who received recommendations on how to handle their type 1 diabetes. For S3, a total of 40.9% (81/197) of the participants were female, 58.1% (114/197) of the participants were male, and 1% (2/197) of the participants did not specify their sex. We gathered a total of 693 participants, who were distributed among the scenarios (a) physician only, (b) physician with AI support, and (c) AI only as follows: S1a: n=101, 14.6%; S1b: n=74, 10.7%; S1c: n=83, 12%; S2a: n=78, 11.3%; S2b: n=83, 12%; S2c: n=77, 11.1%; S3a: n=67, 9.7%; S3b: n=60, 8.7%; and S3c: n=70, 10.1%. The mean average ages were 37.08 (SD 12.07) years for S1 and S2 and 36.04 (SD 11.52) years for S3. Our participants were aged between 18 and 70 years. We further measured some descriptives that included general computer skills (S1 and S2: mean 4.25, SD 0.744 and S3: mean 4.29, SD 0.744); experience with AI-based services (S1 and S2: mean 3.06, SD 0.917 and S3: mean 3.24, SD 0.993); and technical knowledge about AI (S1 and S2: mean 3.34, SD 0.850 and S3: mean 3.47, SD 0.867).

### Validity and Reliability

A partial least squares (PLS) approach to structural equation modeling (SEM) was used to test individuals’ intention to use AI-based HIS short- and long-term medical treatments. We used the Software SmartPLS 4.1.0.4. Variance-based SEM is more suitable than covariance-based SEM in cases when target constructs are to be predicted and explained with the constructs and the main drivers in structural models [70]. For our investigation, we used bootstrapping and permutation procedures with 5000 subsamples using recommendations of Hair et al [70,71] for PLS-SEM for short- and long-term treatments alike. Thus, we had to evaluate our measurement and structural models to ensure that we could compare our scenarios appropriately and report on the predictive power of our PLS-SEM. First, we investigated our reflective measurement models by considering the respective loadings, internal consistency reliability, convergent validity, and discriminant validity. The reflective variables in our investigation are “abilities (HIS),” “intention to use,” “attitude (HIS),” “attitude (disease),” “perceived norms,” “perceived behavior control,” “perceived response efficacy,” “perceived costs/barriers (HIS),” “perceived threat vulnerability,” “fear (HIS),” and “fear (disease).” We determined that all reflective variables and items surpassed the respective thresholds. Second, we examined our formative measurement models considering convergent validity, collinearity, and loadings and weights of our formative items. Our sole formative variable is “perceived threat severity.” The formative variable and items surpassed the respective thresholds. Third, we investigated our structural models by reporting on collinearity and *R*² values to display the explanation power of our study, as well as the standardized root mean square residual [72]. We determined values of 0.087 (S1 and S2) and 0.089 (S3) for our saturated models.

Furthermore, we performed PLSpredict and cross-validated predictive ability test examinations to report on the predictive power of our models and used Measurement Invariance Assessment procedures to report on significant differences between groups. To report on those significances, Hair et al [70,71] stated that *f*² values have to be considered that were adapted from Cohen [73]. According to Cohen [73], *f*² values of 0.02, 0.15, and 0.35 represent small, medium, and large effect sizes, respectively. Furthermore, Kock and Hadaya [74] proposed that *f*² values depend on sample size and therefore need to be adjusted based on the sample used. Accordingly, we determined the following *f*² values for our scenarios by using G*Power (version 3.1.9.7 [75]): S1a: 0.0621478, S1b: 0.0853821, S1c: 0.0759154, S2a: 0.0808975, S2b: 0.0759154, S2c: 0.0819737, S3a: 0.0945622, S3b: 0.105969, and S3c: 0.0903957. By investigating PLSpredict, cross-validated predictive ability test, Measurement Invariance Assessment, and *f*² values, we determined that our integrated theoretical model was sufficient to report on group differences and to predict further investigations toward the intention to use.

### Ethical Considerations

All procedures performed in our survey involving human participants were in accordance with the ethical standards of the 1964 Declaration of Helsinki and its later amendments or comparable ethical standards. As stated, the data were collected with a platform (Clickworker) on which participants contribute anonymously and receive a money award for their time spent, which was about €3 (US $3) per survey. Furthermore, we ensured that our participants provided informed consent, permitting the use of the survey data for research purposes. We ensured that our data analysis and presentation is exclusively anonymized and therefore no conclusions can be drawn about certain individuals. As a consequence, the Ethics Committee of the School of Business and Economics at the University of Marburg evaluated the research design and process and granted an exempt status.

## Results

**Figure 2:**
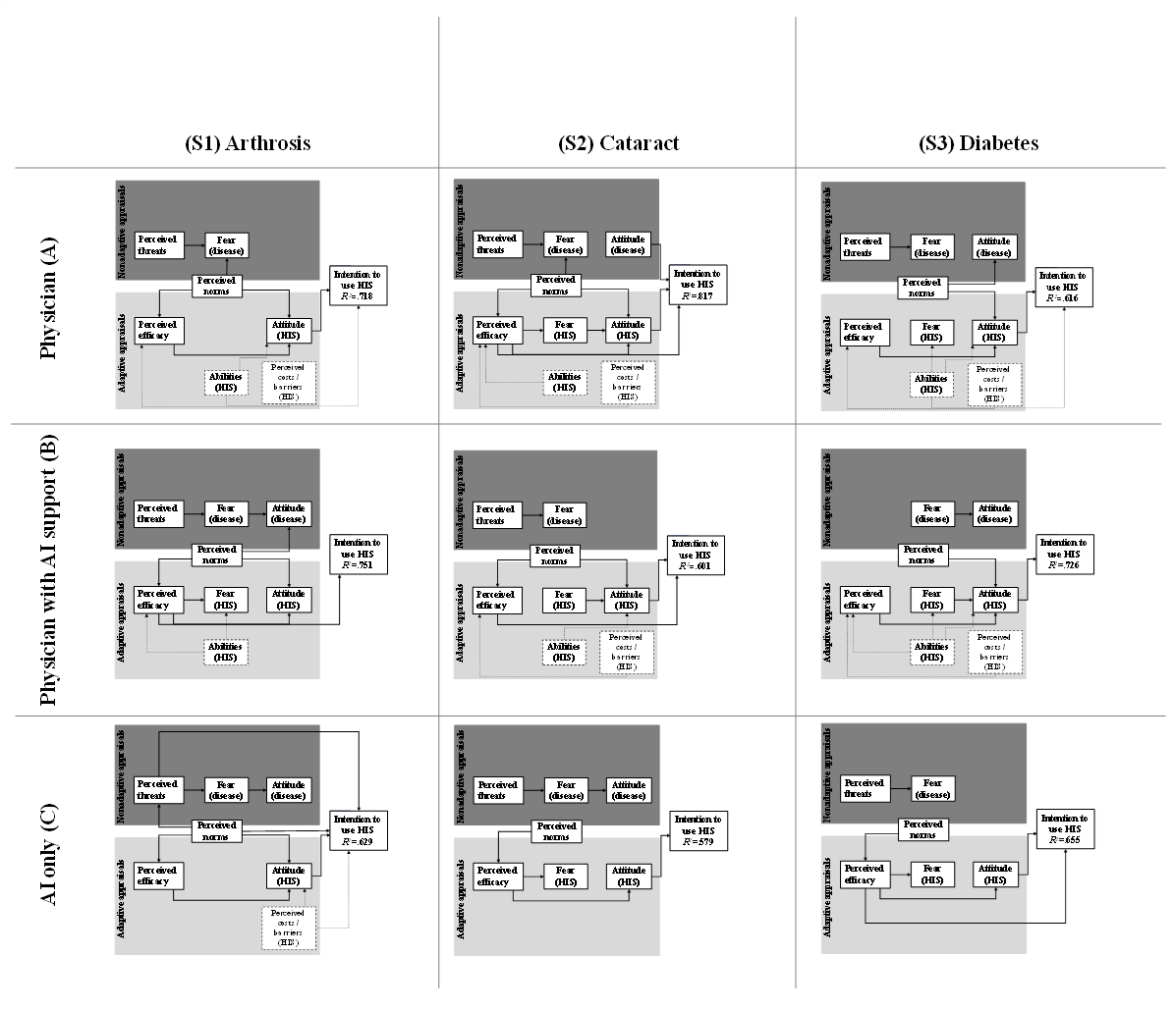
Research model results for the scenarios. HIS: health information systems. For a higher-resolution version of this figure, see Multimedia Appendix 2.

The mean values of the respective intentions are as follows: S1a: 3.72, S1b: 3.42, S1c: 3.12, S2a: 4.24; S2b: 3.69, S2c: 2.79, S3a: 3.92, S3b: 3.87, and S3c: 3.98. Furthermore, we used our integrated theoretical model to estimate the variables that predict the intention to use. Figure 2 provides an overview of all scenarios, highlighting the variables with significant path coefficients only.

According to our integrated theoretical model and scenarios, our *R*² values vary between 57.9% and 81.7%, thus representing medium to good explanation powers of our model [70]. Compared to other *R*² values of other integrated frameworks, such as 42% of Chau and Hu [50], 67% of Ku and Hsieh [17], as well as 45% of Zhang et al [56], we performed quite well with our model. An overview of all path coefficients in the model according to the hypotheses can be found in Multimedia Appendix 1 for each scenario.

## Discussion

### Principal Findings

In our investigation, we designed an integrated model to determine intentions to use AI-based HIS for short- and long-term treatments. Our results revealed that intention to use is well predicted by our model. Furthermore, to answer our research question, we estimated several variables that predict the intention to use for short- and long-term treatments. Thus, our model is sufficient for this and further investigations addressing the intention to use AI-based HIS.

By comparing short- and long-term treatments, our results showed that there were several differences in estimating the significance of our hypotheses. First, perceived threats of fear (disease) are important to consider. Thus, we support the propositions of Gao et al [18], who investigated wearable devices in health care and stated that perceived threats and perceived effectiveness are mandatory for the intention to use HIS. While both of our variables, threats and fear (disease), are significant variables while physicians participate in the treatment, threats are less important when the treatment is performed by AI only. In our case, treating arthrosis, cataracts, and diabetes by physicians only (or supported by AI-based HIS) are treatments that are known to individuals. We conclude that treating such diseases by humans is the most common appearance in individuals’ perceptions of surgeries [34]. Furthermore, we conclude that nonadaptive appraisals are more important when the treatment is known. We do so as we support the propositions of Longoni et al [1], who stated that individuals are more likely to rely on recommendations from physicians than from AI. According to them, physicians perform better than AI in evaluating an individual’s medical history. In contrast, adaptive appraisals are more important when the treatment is unknown to individuals, for example, as it is supported by AI only. This also matches with further differences in the significance we determined with our integrated model.

Second, perceived efficacy toward attitude (HIS) is important for all scenarios. Hence, we support the proposition of Venkatesh et al [49], who stated that perceived efficacy is one of the major predictors of determining the intention to use new information systems. Furthermore, Palmisciano et al [30] supported this proposition as they investigated the attitudes of patients and their relatives concerning the use of AI in neurosurgery. They stated that the efficacy individuals perceive toward the efficiency of AI-based HIS is dependent on their attitude toward the HIS.

Third, our results revealed that attitude (HIS) is important to determine the intention to use AI-based HIS. Thus, we support the propositions of Fishbein and Ajzen [41], who stated that attitude is one of the major predictors in determining the intention to use toward a certain behavior. In addition, Esmaeilzadeh [7] supports the propositions of Fishbein and Ajzen [41] and Palmisciano et al [30], as she estimated that attitude and perceived effectiveness are important for the intention to use. Furthermore, physicians are relevant with regard to the intention to use AI-based HIS; for example, training leads to more positive attitudes toward HIS and is beneficial for the intention to use. Furthermore, our proposition is supported by Yakar et al [35], who determined that attitude toward AI is important for intention to use in a Dutch society. In contrast to our study, their participants actually stayed in a Dutch hospital and were surveyed concerning treatments in radiology, robotic surgery, and dermatology. Hence, and for our propositions concerning arthrosis and cataracts, attitudes might change if individuals actually fall ill with the relevant diseases, which might also lead to changes in their behavioral intention. Fourth, we determined that perceived norms are important in our integrated model, as we estimated the significance of attitude (HIS) as well as perceived efficacy. Hence, we support the propositions of Broadbent et al [32] and Dziergwa et al [12]. While Broadbent et al [32] determined that the acceptance of social care robots by older adults was low in 2009, Dziergwa et al [12] estimated the opposite in 2017, reflecting high acceptance by older adults in 2017. They stated that society plays an important role in the perception of new information systems and that this perception may vary over time due to experiences. As such, the acceptance of social care robots changed in society over time, reflecting that perceived norms as opinions of others are important in the determination of intention to use and might lead to a change in perception. Fifth, our results showed that once AI is involved in our treatments, fear toward HIS is important, while fear toward the disease is important for all scenarios. Hence, our results support the propositions of Yun et al [36] and Fritsch et al [40] that emotions are important when determining the intention to use medical treatments by both physicians and AI. In accordance with Yun et al [36] and Fritsch et al [40], fear (HIS) is important for all scenarios with AI involvement. Hence, we conclude that our participants are concerned about whether AI is able to perform proper treatments. Sixth, we determined that perceived efficacy toward intention to use differs in significance between short- and long-term treatments. While significance was determined for treatments by physicians and physicians with AI support concerning short-term treatments, it is not significant for long-term treatments. In contrast, perceived efficacy toward intention to use is significant for long-term treatments performed by AI only, while it is not significant for short-term treatments. Hence, our results support the propositions of Palmisciano et al [30] and Esmaeilzadeh [7], who stated that patients want physicians to monitor autonomous AI treatments as they doubt the efficacy of AI-based HIS.

### Theoretical Implications

Our study leads to several theoretical implications. First, we propose an integrated theoretical model that is helpful for the explanation and prediction of the intention to use AI-based HIS. With positive results of various tests concerning measurement and structural models as well as model predictions, we recommend that our model be used in future studies that address the intention to use AI-based HIS. Compared to models from information systems, the prediction gets better due to the duality approach. In comparison to the health care models, we add the specifics of information systems, which enhance the duality approach beyond general treatments. Second, our integrated model benefits the theories we addressed from health care, information systems, and psychology. In particular, we show how adaptive and nonadaptive appraisals are balanced when individuals determine the intention to use information system–based medical treatments that are unknown and not available yet. Third, our empirical results contribute to research that addresses short- and long-term medical treatments. We provide certain scenarios that can be used in other studies to estimate the intention to use AI-based HIS. Fourth, we benefit from studies that address different technologies, that is, concerning different types of AI involvement in medical treatments. In particular, we show how the intention to use medical treatments differs when AI is not or only partly existent or performs the treatment on its own.

### Practical Implications

Our results have several practical implications. First, health care professionals and physicians can use our results in their daily work. As we determined that perceived efficacy and attitude (HIS) are mandatory for the intention to use AI-based HIS, we propose that health care professionals and physicians consider those variables when advertising novel HIS-based treatments. In particular, they should address information that influences attitude (HIS) positively and raise perceived efficacy in that they clarify risks and show the benefits of AI-based treatments. Second, information system professionals benefit from our study, as we investigated patients as important stakeholders in AI-based HIS. Specifically, we estimated that many patients intend to use AI-based HIS and thus support the propositions of Tran et al [31] that the demand for AI-based HIS is high. Thus, we propose that information system professionals consider fear (HIS) in their development, as they should—combined with perceived efficacy—ensure that AI-based HIS are error-prone. Then, error-prone HIS should lower fear (HIS) and improve perceived efficacy. Third, our results benefit health care management. As we ascertained that the overall perception of AI-based HIS in society changes over time and thus acceptance is rising, we propose that management performs AI training. In particular, health care management should organize training for health care professionals and physicians to improve treatments that are supported or performed by AI-based HIS. Ultimately, such training should lower error rates and hence lead to more positive attitudes toward such HIS and higher perceived efficacies.

### Limitations and Future Work

Our study has some limitations. First, we investigated scenario descriptions for our study and our participants for short-term treatments, and our participants had to imagine that they fell ill with the disease. Thus, in contrast to our long-term treatments, our participants did not have arthrosis and cataracts, and their behavioral intention might change when they actually fall ill. Nonetheless, other studies show that attitude is important for individuals who actually stay in a hospital [35]. Second, the participants in our empirical study had an average experience with AI and new technologies in general, while we acquired individuals from across German society. Although these variables had no significant impact, groups with more or less experience could lead to different results than ours. Other studies from Germany determined similar results from a patient point of view [40], while AI is perceived better by German physicians, who had better abilities toward AI use [39]. Furthermore, recruiting participants from other cultural backgrounds could lead to different results. Third, our investigation is subject to the famous intention-behavior gap [41]. Although intention is the most important to predict human behavior, individuals might act differently in their actual behavior, as there might be other variables that influence their behavior. Hence, future studies should address various factors that influence intention to use AI. They could use duality approaches when patients are actually skilled in AI use due to various utilizations for medical purposes instead of using hypothetical scenarios like in this study. Furthermore, combined studies would be interesting to consider existing fear toward AI-based HIS. Those studies could then investigate how AI-based HIS perform while using augmented reality to reduce pain as proposed by Ahmadpour et al [37] and Spiegel [38] simultaneously.

### Conclusions

We investigated short- and long-term medical treatments with AI-based HIS using an integrated theoretical model to predict human behavior. To do so, we draw on theories from health care, information systems, and psychology. On the basis of the high explanation powers and significant variables that we determined, we conclude that our integrated theoretical model is sufficient for such investigations. Furthermore, we answer our research question by determining several factors that significantly influence the intention to use AI-based HIS for both adaptive and nonadaptive appraisals. Hence, individuals indeed balance between adaptive and nonadaptive appraisals when determining the intention to use new health care inventions. While nonadaptive appraisals are important when the treatment is known, adaptive appraisals are mandatory when the treatment is unknown. As key takeaways, perceived efficacy and attitude (HIS) are important factors to determine the intention to use AI-based HIS. Hence, we conclude that future AI-based HIS developments and research should specifically address perceived efficacy as well as attitude (HIS) of new AI-based HIS. Furthermore, our integrated model should be used to investigate the intention to use AI-based HIS in the future. Ultimately, we derive theoretical and practical implications, show future research directions, and discuss the limitations of our study that we have presented in the previous sections.

## Appendix

Multimedia Appendix 1 Correlation tables for all scenarios as well as the path coefficients concerning the study hypotheses.

Multimedia Appendix 2 Research model results for the scenarios. HIS: health information systems.

## Abbreviations

AI: artificial intelligence
EPPM: Extended Parallel Process Model
HBM: Health Belief Model
HIS: health information systems
PLS: partial least squares
PMT: Protection Motivation Theory
RAA: reasoned action approach
SEM: structural equation modeling
TAM: Technology Acceptance Model
TPB: Theory of Planned Behavior
UTAUT2: Unified Theory of Acceptance and Use of Technology 2

## Data Availability

The study data are available on the internet [76]. The following files for all scenarios are provided: main analyses, questionnaires, scenarios, bootstrap, permutation analyses, partial least squares algorithm, PLSpredict, *f*² values.
