# Supplementary material for: Acceptance of AI in Health Care for Short- and Long-Term Treatments: Pilot Development Study of an Integrated Theoretical Model
Source: JMIR Form Res. 2024 Jul 18;8:e48600. doi: 10.2196/48600 (PMC11294784; doi:10.2196/48600)
Supplement: Multimedia Appendix 1 [file formative_v8i1e48600_app1.docx]

Table S1. Correlations concerning the survey Arthrosis Physician, n = 101, location = Germany, time frame= April 2021

|  | 1 | 2 | 3 | 4 | 5 | 6 | 7 | 8 | 9 | 10 | 11 | 12 | 13 |
| --- | --- | --- | --- | --- | --- | --- | --- | --- | --- | --- | --- | --- | --- |
| 1 | 1 | .812  (*P*<.001) | -.394  (*P*<.001) | .550  (*P*<.001) | .596  (*P*<.001) | .450  (*P*<.001) | -.285  (*P*=.004) | .009  (*P*=.929) | .038  (*P*=.708) | .117  (*P*=.245) | -.420  (*P*<.001) | .065  (*P*=.515) | .290  (*P*=.003) |
| 2 |  | 1 | -.386  (*P*<.001) | .618  (*P*<.001) | .647  (*P*<.001) | .413  (*P*<.001) | -.244  (*P*=.014) | .031  (*P*=.757) | .127  (*P*=.206) | .293  (*P*=.003) | -.397  (*P*<.001) | .079  (*P*=.435) | .278  (*P*=.005) |
| 3 |  |  | 1 | -.266  (*P*=.007) | -.286  (*P*=.004) | -.369  (*P*<.001) | .402  (*P*<.001) | -.055  (*P*=.583) | .049  (*P*=.626) | .033  (*P*=.745) | .308  (*P*=.002) | -.162  (*P*=.106) | .223  (*P*=.025) |
| 4 |  |  |  | 1 | .500  (*P*<.001) | .314  (*P*=.001) | -.136  (*P*=.176) | .181  (*P*=.071) | .032  (*P*=.748) | .222  (*P*=.026) | -.234  (*P*=.019) | .037  (*P*=.717) | .335  (*P*<.001) |
| 5 |  |  |  |  | 1 | .309  (*P*=.002) | -.188  (*P*=.059) | .143  (*P*=.153) | -.025  (*P*=.804) | .248  (*P*=.012) | -.421  (*P*<.001) | .058  (*P*=.564) | .249  (*P*=.012) |
| 6 |  |  |  |  |  | 1 | -.261  (*P*=.008) | .087  (*P*=.388) | .241  (*P*=.015) | .117  (*P*=.242) | -.219  (*P*=.027) | -.071  (*P*=.478) | .041  (*P*=.682) |
| 7 |  |  |  |  |  |  | 1 | -.071  (*P*=.481) | .140  (*P*=.757) | .040  (*P*=.693) | .340  (*P*<.001) | -.110  (*P*=.275) | .135  (*P*=.178) |
| 8 |  |  |  |  |  |  |  | 1 | -.135  (*P*=.178) | -.220  (*P*=.027) | -.180  (*P*=.071) | .357  (*P*<.001) | -.095  (*P*=.343) |
| 9 |  |  |  |  |  |  |  |  | 1 | .411  (*P*<.001) | .164  (*P*=.100) | -.225  (*P*=.024) | .090  (*P*=.370) |
| 10 |  |  |  |  |  |  |  |  |  | 1 | -.049  (*P*=.630) | -.127  (*P*=.204) | .363  (*P*<.001) |
| 11 |  |  |  |  |  |  |  |  |  |  | 1 | -.162  (*P*=.105) | -.056  (*P*=.578) |
| 12 |  |  |  |  |  |  |  |  |  |  |  | 1 | -.015  (*P*=.878) |
| 13 |  |  |  |  |  |  |  |  |  |  |  |  | 1 |

Table S2. Correlations concerning the survey Arthrosis Physician with AI-support, n = 74, location = Germany, time frame= April 2021

|  | 1 | 2 | 3 | 4 | 5 | 6 | 7 | 8 | 9 | 10 | 11 | 12 | 13 |
| --- | --- | --- | --- | --- | --- | --- | --- | --- | --- | --- | --- | --- | --- |
| 1 | 1 | .872  (*P*<.001) | -.559  (*P*<.001) | .680  (*P*<.001) | .695  (*P*<.001) | .423  (*P*<.001) | -.177  (*P*=.132) | -.058  (*P*=.625) | -.187  (*P*=.110) | -.028  (*P*=.815) | -.234  (*P*=.045) | -.043  (*P*=.716) | .174  (*P*=.139) |
| 2 |  | 1 | -.569  (*P*<.001) | .665  (*P*<.001) | .697  (*P*<.001) | .481  (*P*<.001) | -.203  (*P*=.082) | -.056  (*P*=.638) | -.169  (*P*=.150) | -.030  (*P*=.799) | -.234  (*P*=.045) | .048  (*P*=.682) | .236  (*P*=.043) |
| 3 |  |  | 1 | -.361  (*P*=.002) | -.439  (*P*<.001) | -.323  (*P*=.005) | .367  (*P*=.001) | .122  (*P*=.299) | .294  (*P*=.011) | .153  (*P*=.194) | .330  (*P*=.004) | .146  (*P*=.214) | -.065  (*P*=.585) |
| 4 |  |  |  | 1 | .572  (*P*<.001) | .400  (*P*<.001) | -.065  (*P*=.582) | -.008  (*P*=.943) | .026  (*P*=.828) | .195  (*P*=.097) | .046  (*P*=.699) | -.048  (*P*=.684) | .354  (*P*=.002) |
| 5 |  |  |  |  | 1 | .410  (*P*<.001) | -.268  (*P*=.021) | .048  (*P*=.682) | -.250  (*P*=.032) | -.079  (*P*=.505) | -.363  (*P*=.001) | .249  (*P*=.033) | .060  (*P*=.609) |
| 6 |  |  |  |  |  | 1 | .134  (*P*=.255) | -.027  (*P*=.820) | -.072  (*P*=.540) | -.120  (*P*=.308) | .152  (*P*=.196) | -.139  (*P*=.239) | -.069  (*P*=.557) |
| 7 |  |  |  |  |  |  | 1 | -.050  (*P*=.673) | .157  (*P*=.181) | -.108  (*P*=.358) | .544  (*P*<.001) | -.188  (*P*=.109) | -.225  (*P*=.054) |
| 8 |  |  |  |  |  |  |  | 1 | -.028  (*P*=.810) | .015  (*P*=.902) | -.013  (*P*=.914) | .355  (*P*=.002) | -.057  (*P*=.629) |
| 9 |  |  |  |  |  |  |  |  | 1 | .657  (*P*<.001) | .488  (*P*<.001) | -.143  (*P*=.225) | .452  (*P*<.001) |
| 10 |  |  |  |  |  |  |  |  |  | 1 | .231  (*P*=.048) | .020  (*P*=.865) | .609  (*P*<.001) |
| 11 |  |  |  |  |  |  |  |  |  |  | 1 | -.218  (*P*=.062) | .170  (*P*=.148) |
| 12 |  |  |  |  |  |  |  |  |  |  |  | 1 | .002  (*P*=.988) |
| 13 |  |  |  |  |  |  |  |  |  |  |  |  | 1 |

Table S3. Correlations concerning the survey Arthrosis AI, n = 83, location = Germany, time frame= April 2021

|  | 1 | 2 | 3 | 4 | 5 | 6 | 7 | 8 | 9 | 10 | 11 | 12 | 13 |
| --- | --- | --- | --- | --- | --- | --- | --- | --- | --- | --- | --- | --- | --- |
| 1 | 1 | .731  (*P*<.001) | -.558  (*P*<.001) | .440  (*P*<.001) | .631  (*P*<.001) | .594  (*P*<.001) | -.272  (*P*=.013) | .100  (*P*=.368) | -.007  (*P*=.950) | .011  (*P*=.923) | -.107  (*P*=.336) | -.179  (*P*=.105) | .086  (*P*=.442) |
| 2 |  | 1 | -.554  (*P*<.001) | .592  (*P*<.001) | .674  (*P*<.001) | .576  (*P*<.001) | -.283  (*P*=.010) | .197  (*P*=.074) | -.052  (*P*=.638) | .043  (*P*=.698) | -.119  (*P*=.285) | -.153  (*P*=.168) | .152  (*P*=.169) |
| 3 |  |  | 1 | -.377  (*P*<.001) | -.390  (*P*<.001) | -.451  (*P*<.001) | .285  (*P*=.009) | .023  (*P*=.839) | .241  (*P*=.028) | .063  (*P*=.572) | .049  (*P*=.659) | .043  (*P*=.699) | .129  (*P*=.245) |
| 4 |  |  |  | 1 | .325  (*P*=.003) | .332  (*P*=.002) | -.142  (*P*=.199) | .097  (*P*=.383) | .039  (*P*=.724) | .156  (*P*=.159) | -.019  (*P*=.862) | -.326  (*P*=.003) | .046  (*P*=.682) |
| 5 |  |  |  |  | 1 | .400  (*P*<.001) | -.457  (*P*<.001) | .417  (*P*<.001) | -.081  (*P*=.469) | -.018  (*P*=.869) | -.420  (*P*<.001) | .083  (*P*=.453) | .169  (*P*=.127) |
| 6 |  |  |  |  |  | 1 | -.204  (*P*=.065) | -.034  (*P*=.763) | .255  (*P*=.020) | .270  (*P*=.014) | .100  (*P*=.370) | -.243  (*P*=.027) | .319  (*P*=.003) |
| 7 |  |  |  |  |  |  | 1 | -.051  (*P*=.644) | .168  (*P*=.130) | .110  (*P*=.320) | .593  (*P*<.001) | -.122  (*P*=.273) | -.010  (*P*=.928) |
| 8 |  |  |  |  |  |  |  | 1 | -.024  (*P*=.829) | .039  (*P*=.723) | -.163  (*P*=.141) | .240  (*P*=.029) | .081  (*P*=.465) |
| 9 |  |  |  |  |  |  |  |  | 1 | .602  (*P*<.001) | .119  (*P*=.284) | -.283  (*P*=.010) | .396  (*P*<.001) |
| 10 |  |  |  |  |  |  |  |  |  | 1 | .059  (*P*=.596) | -.225  (*P*=.041) | .553  (*P*<.001) |
| 11 |  |  |  |  |  |  |  |  |  |  | 1 | -.135  (*P*=.223) | .075  (*P*=.502) |
| 12 |  |  |  |  |  |  |  |  |  |  |  | 1 | -.156  (*P*=.160) |
| 13 |  |  |  |  |  |  |  |  |  |  |  |  | 1 |

Table S4. Correlations concerning the survey Cataract Physician, n = 78, location = Germany, time frame= April 2021

|  | 1 | 2 | 3 | 4 | 5 | 6 | 7 | 8 | 9 | 10 | 11 | 12 | 13 |
| --- | --- | --- | --- | --- | --- | --- | --- | --- | --- | --- | --- | --- | --- |
| 1 | 1 | .667  (*P*<.001) | -.351  (*P*=.002) | .449  (*P*<.001) | .854  (*P*<.001) | .368  (*P*<.001) | -.181  (*P*=.112) | .215  (*P*=.058) | -.170  (*P*=.137) | -.214  (*P*=.061) | -.292  (*P*=.009) | .268  (*P*=.017) | -.010  (*P*=.930) |
| 2 |  | 1 | -.367  (*P*<.001) | .587  (*P*<.001) | .712  (*P*<.001) | .473  (*P*<.001) | -.246  (*P*=.030) | .239  (*P*=.035) | -.024  (*P*=.837) | -.312  (*P*=.005) | -.240  (*P*=.035) | .464  (*P*<.001) | -.009  (*P*=.936) |
| 3 |  |  | 1 | -.032  (*P*=.783) | -.302  (*P*=.007) | -.198  (*P*=.082) | .520  (*P*<.001) | -.153  (*P*=.180) | .139  (*P*=.225) | .289  (*P*=.010) | .423  (*P*<.001) | .090  (*P*=.435) | .393  (*P*<.001) |
| 4 |  |  |  | 1 | .473  (*P*<.001) | .324  (*P*=.004) | .001  (*P*=.991) | .210  (*P*=.064) | -.103  (*P*=.368) | -.157  (*P*=.171) | -.149  (*P*=.192) | .579  (*P*<.001) | .053  (*P*=.646) |
| 5 |  |  |  |  | 1 | .429  (*P*<.001) | -.187  (*P*=.100) | .161  (*P*=.159) | -.044  (*P*=.703) | -.256  (*P*=.024) | -.230  (*P*=.043) | .361  (*P*=.001) | -.017  (*P*=.884) |
| 6 |  |  |  |  |  | 1 | -.263  (*P*=.020) | .293  (*P*=.009) | .027  (*P*=.812) | -.231  (*P*=.042) | -.086  (*P*=.455) | .292  (*P*=.009) | -.148  (*P*=.195) |
| 7 |  |  |  |  |  |  | 1 | -.044  (*P*=.699) | .062  (*P*=.592) | .222  (*P*<.001) | .437  (*P*<.001) | .161  (*P*=.158) | .321  (*P*=.004) |
| 8 |  |  |  |  |  |  |  | 1 | -.050  (*P*=.662) | -.209  (*P*=.067) | -.196  (*P*=.085) | .344  (*P*=.002) | .129  (*P*=.262) |
| 9 |  |  |  |  |  |  |  |  | 1 | .251  (*P*=.027) | .320  (*P*=.004) | .215  (*P*=.059) | .226  (*P*=.046) |
| 10 |  |  |  |  |  |  |  |  |  | 1 | .146  (*P*=.202) | -.053  (*P*=.642) | .388  (*P*<.001) |
| 11 |  |  |  |  |  |  |  |  |  |  | 1 | .102  (*P*=.373) | .044  (*P*=.703) |
| 12 |  |  |  |  |  |  |  |  |  |  |  | 1 | .303  (*P*=.007) |
| 13 |  |  |  |  |  |  |  |  |  |  |  |  | 1 |

Table S5. Correlations concerning the survey Cataract Physician with AI support, n = 83, location = Germany, time frame= April 2021

|  | 1 | 2 | 3 | 4 | 5 | 6 | 7 | 8 | 9 | 10 | 11 | 12 | 13 |
| --- | --- | --- | --- | --- | --- | --- | --- | --- | --- | --- | --- | --- | --- |
| 1 | 1 | .682  (*P*<.001) | -.475  (*P*<.001) | .578  (*P*<.001) | .483  (*P*<.001) | .505  (*P*<.001) | -.195  (*P*=.078) | .218  (*P*=.048) | -.103  (*P*=.355) | -.040  (*P*=.722) | -.305  (*P*=.005) | -.008  (*P*=.946) | -.114  (*P*=.304) |
| 2 |  | 1 | -.562  (*P*<.001) | .603  (*P*<.001) | .423  (*P*<.001) | .639  (*P*<.001) | -.254  (*P*=.020) | .156  (*P*=.158) | -.053  (*P*=.634) | -.049  (*P*=.662) | -.365  (*P*<.001) | .069  (*P*=.534) | -.069  (*P*=.537) |
| 3 |  |  | 1 | -.311  (*P*=.004) | -.341  (*P*=.002) | -.307  (*P*=.005) | .307  (*P*=.005) | -.079  (*P*=.478) | .244  (*P*=.026) | .158  (*P*=.153) | .302  (*P*=.005) | .000  (*P*=.998) | .227  (*P*=.039) |
| 4 |  |  |  | 1 | .461  (*P*<.001) | .554  (*P*<.001) | -.188  (*P*=.089) | .301  (*P*=.006) | .167  (*P*<.001) | .140  (*P*=.208) | -.196  (*P*=.076) | .145  (*P*=.192) | .074  (*P*=.509) |
| 5 |  |  |  |  | 1 | .284  (*P*=.009) | -.371  (*P*<.001) | .386  (*P*<.001) | -.209  (*P*=.059) | -.191  (*P*=.084) | -.370  (*P*<.001) | .171  (*P*=.123) | .028  (*P*=.800) |
| 6 |  |  |  |  |  | 1 | -.217  (*P*=.049) | .244  (*P*=.026) | .085  (*P*=.447) | .027  (*P*=.811) | -.304  (*P*=.005) | .072  (*P*=.518) | -.052  (*P*=.640) |
| 7 |  |  |  |  |  |  | 1 | -.275  (*P*=.012) | .332  (*P*=.002) | .204  (*P*=.064) | .504  (*P*<.001) | -.383  (*P*<.001) | .003  (*P*=.978) |
| 8 |  |  |  |  |  |  |  | 1 | -.187  (*P*=.091) | -.149  (*P*=.180) | -.243  (*P*=.027) | .358  (*P*<.001) | -.101  (*P*=.365) |
| 9 |  |  |  |  |  |  |  |  | 1 | .434  (*P*<.001) | .187  (*P*=.090) | -.049  (*P*=.662) | .147  (*P*=.183) |
| 10 |  |  |  |  |  |  |  |  |  | 1 | .206  (*P*=.062) | .098  (*P*=.376) | .289  (*P*=.008) |
| 11 |  |  |  |  |  |  |  |  |  |  | 1 | -.271  (*P*=.013) | -.009  (*P*=.938) |
| 12 |  |  |  |  |  |  |  |  |  |  |  | 1 | -.014  (*P*=.900) |
| 13 |  |  |  |  |  |  |  |  |  |  |  |  | 1 |

Table S6. Correlations concerning the survey Cataract AI, n = 77, location = Germany, time frame= April 2021

|  | 1 | 2 | 3 | 4 | 5 | 6 | 7 | 8 | 9 | 10 | 11 | 12 | 13 |
| --- | --- | --- | --- | --- | --- | --- | --- | --- | --- | --- | --- | --- | --- |
| 1 | 1 | .809  (*P*<.001) | -.612  (*P*<.001) | .577  (*P*<.001) | .524  (*P*<.001) | .650  (*P*<.001) | -.006  (*P*=.960) | .109  (*P*=.344) | -.114  (*P*=.323) | .053  (*P*=.650) | -.300  (*P*=.008) | -.022  (*P*=.849) | -.065  (*P*=.575) |
| 2 |  | 1 | -.664  (*P*<.001) | .520  (*P*<.001) | .570  (*P*<.001) | .643  (*P*<.001) | -.228  (*P*=.046) | .164  (*P*=.154) | -.163  (*P*=.156) | -.070  (*P*=.545) | -.459  (*P*<.001) | .017  (*P*=.884) | -.193  (*P*=.092) |
| 3 |  |  | 1 | -.358  (*P*<.001) | -.292  (*P*=.010) | -.532  (*P*<.001) | .203  (*P*=.077) | .028  (*P*=.807) | .109  (*P*=.345) | .202  (*P*=.078) | .416  (*P*<.001) | -.023  (*P*=.841) | .286  (*P*=.012) |
| 4 |  |  |  | 1 | .290  (*P*=.010) | .449  (*P*<.001) | .178  (*P*=.121) | -.060  (*P*=.603) | .092  (*P*=.426) | .273  (*P*=.016) | .099  (*P*=.392) | -.165  (*P*=.153) | .034  (*P*=.767) |
| 5 |  |  |  |  | 1 | .443  (*P*<.001) | -.307  (*P*=.007) | .435  (*P*<.001) | -.305  (*P*=.007) | -.191  (*P*=.096) | -.504  (*P*<.001) | .324  (*P*=.004) | .028  (*P*=.810) |
| 6 |  |  |  |  |  | 1 | -.046  (*P*=.691) | .062  (*P*=.590) | -.220  (*P*=.054) | .012  (*P*=.916) | -.211  (*P*=.065) | -.073  (*P*=.530) | -.244  (*P*=.033) |
| 7 |  |  |  |  |  |  | 1 | -.235  (*P*=.040) | .143  (*P*=.216) | .234  (*P*=.041) | .641  (*P*<.001) | -.177  (*P*=.123) | .115  (*P*=.317) |
| 8 |  |  |  |  |  |  |  | 1 | -.160  (*P*=.165) | -.029  (*P*=.805) | -.329  (*P*=.003) | .330  (*P*=.003) | .060  (*P*=.601) |
| 9 |  |  |  |  |  |  |  |  | 1 | .521  (*P*<.001) | .220  (*P*=.055) | -.219  (*P*=.056) | -.026  (*P*=.820) |
| 10 |  |  |  |  |  |  |  |  |  | 1 | ,298  (*P*=.009) | -.265  (*P*=.020) | .266  (*P*=.019) |
| 11 |  |  |  |  |  |  |  |  |  |  | 1 | -.321  (*P*=.004) | .184  (*P*=.108) |
| 12 |  |  |  |  |  |  |  |  |  |  |  | 1 | .303  (*P*=.007) |
| 13 |  |  |  |  |  |  |  |  |  |  |  |  | 1 |

Table S7. Correlations concerning the survey Diabetes Physician, n = 67, location = Germany, time frame= May 2021

|  | 1 | 2 | 3 | 4 | 5 | 6 | 7 | 8 | 9 | 10 | 11 | 12 | 13 |
| --- | --- | --- | --- | --- | --- | --- | --- | --- | --- | --- | --- | --- | --- |
| 1 | 1 | .581  (*P*<.001) | -.219  (*P*=.074) | .639  (*P*<.001) | -.196  (*P*=.113) | .115  (*P*=.353) | .379  (*P*=.002) | .191  (*P*=.121) | .264  (*P*=.031) | .127  (*P*=.307) | -.267  (*P*=.029) | -.035  (*P*=.781) | .263  (*P*=.031) |
| 2 |  | 1 | -.499  (*P*<.001) | .510  (*P*<.001) | -.428  (*P*<.001) | .303  (*P*=.013) | .521  (*P*<.001) | .557  (*P*<.001) | -.005  (*P*=.968) | -.161  (*P*=.193) | -.302  (*P*=.013) | .358  (*P*=.003) | .049  (*P*=.693) |
| 3 |  |  | 1 | -.070  (*P*=.571) | .744  (*P*<.001) | -.029  (*P*=.814) | -.161  (*P*=.194) | -.278  (*P*=.023) | .391  (*P*=.001) | .560  (*P*<.001) | .472  (*P*<.001) | -.163  (*P*=.186) | .390  (*P*=.001) |
| 4 |  |  |  | 1 | .035  (*P*=.776) | .341  (*P*=.005) | .526  (*P*<.001) | .207  (*P*=.093) | .348  (*P*=.004) | .286  (*P*=.019) | -.194  (*P*=.115) | .052  (*P*=.677) | .335  (*P*=.006) |
| 5 |  |  |  |  | 1 | .052  (*P*=.676) | -.073  (*P*=.556) | -.176  (*P*=.155) | .416  (*P*<.001) | .588  (*P*<.001) | .548  (*P*<.001) | -.138  (*P*=.266) | .480  (*P*<.001) |
| 6 |  |  |  |  |  | 1 | .530  (*P*<.001) | .021  (*P*=.865) | .167  (*P*=.177) | .050  (*P*=.685) | .111  (*P*=.371) | .103  (*P*=.407) | .011  (*P*=.927) |
| 7 |  |  |  |  |  |  | 1 | .346  (*P*=.004) | .244  (*P*=.047) | .068  (*P*=.587) | -.016  (*P*=.896) | .104  (*P*=.403) | .120  (*P*=.334) |
| 8 |  |  |  |  |  |  |  | 1 | -.028  (*P*=.819) | -.152  (*P*=.220) | -.163  (*P*=.188) | .359  (*P*=.003) | -.025  (*P*=.840) |
| 9 |  |  |  |  |  |  |  |  | 1 | .716  (*P*<.001) | .153  (*P*=.217) | .057  (*P*=.645) | .665  (*P*<.001) |
| 10 |  |  |  |  |  |  |  |  |  | 1 | .343  (*P*=.004) | .092  (*P*=.457) | .717  (*P*<.001) |
| 11 |  |  |  |  |  |  |  |  |  |  | 1 | .149  (*P*=.230) | .201  (*P*=.103) |
| 12 |  |  |  |  |  |  |  |  |  |  |  | 1 | .303  (*P*=.013) |
| 13 |  |  |  |  |  |  |  |  |  |  |  |  | 1 |

Table S8. Correlations concerning the survey Diabetes Physician with AI-support, n = 60, location = Germany, time frame= May 2021

|  | 1 | 2 | 3 | 4 | 5 | 6 | 7 | 8 | 9 | 10 | 11 | 12 | 13 |
| --- | --- | --- | --- | --- | --- | --- | --- | --- | --- | --- | --- | --- | --- |
| 1 | 1 | .658  (*P*<.001) | -.035  (*P*=.792) | .528  (*P*<.001) | .512  (*P*<.001) | -.149  (*P*=.256) | .613  (*P*<.001) | .615  (*P*<.001) | .184  (*P*=.160) | .148  (*P*=.260) | .010  (*P*=.942) | .027  (*P*=.836) | .044  (*P*=.739) |
| 2 |  | 1 | -.256  (*P*=.049) | .528  (*P*<.001) | .308  (*P*=.017) | -.305  (*P*=.018) | .669  (*P*<.001) | .721  (*P*<.001) | .099  (*P*=.452) | .045  (*P*=.732) | -.091  (*P*=.491) | .233  (*P*=.073) | -.039  (*P*=.766) |
| 3 |  |  | 1 | .032  (*P*=.809) | .121  (*P*=.356) | .635  (*P*<.001) | -.036  (*P*=.785) | -.147  (*P*=.263) | .387  (*P*=.002) | .588  (*P*<.001) | .638  (*P*<.001) | -.108  (*P*=.412) | .561  (*P*<.001) |
| 4 |  |  |  | 1 | .496  (*P*<.001) | -.133  (*P*=.310) | .707  (*P*<.001) | .652  (*P*<.001) | .270  (*P*=.037) | .202  (*P*=.121) | .041  (*P*=.757) | .134  (*P*=.307) | .134  (*P*=.307) |
| 5 |  |  |  |  | 1 | .196  (*P*=.133) | .489  (*P*<.001) | .413  (*P*<.001) | .581  (*P*<.001) | .442  (*P*<.001) | .319  (*P*=.013) | .009  (*P*=.944) | .195  (*P*=.135) |
| 6 |  |  |  |  |  | 1 | -.186  (*P*=.155) | -.188  (*P*<.001) | .449  (*P*<.001) | .450  (*P*<.001) | .642  (*P*<.001) | -.343  (*P*=.007) | .155  (*P*=.236) |
| 7 |  |  |  |  |  |  | 1 | .678  (*P*<.001) | .325  (*P*=.011) | .225  (*P*=.085) | .022  (*P*=.866) | .042  (*P*=.753) | .101  (*P*=.444) |
| 8 |  |  |  |  |  |  |  | 1 | .255  (*P*=.050) | .043  (*P*=.745) | -.070  (*P*=.594) | .252  (*P*=.052) | -.005  (*P*=.970) |
| 9 |  |  |  |  |  |  |  |  | 1 | .577  (*P*<.001) | .510  (*P*<.001) | -.128  (*P*=.331) | .423  (*P*<.001) |
| 10 |  |  |  |  |  |  |  |  |  | 1 | .572  (*P*<.001) | .071  (*P*=.590) | .697  (*P*<.001) |
| 11 |  |  |  |  |  |  |  |  |  |  | 1 | -.159  (*P*=.224) | .331  (*P*=.010) |
| 12 |  |  |  |  |  |  |  |  |  |  |  | 1 | .361  (*P*=.005) |
| 13 |  |  |  |  |  |  |  |  |  |  |  |  | 1 |

Table S9. Correlations concerning the survey Diabetes AI, n = 70, location = Germany, time frame= May 2021

|  | 1 | 2 | 3 | 4 | 5 | 6 | 7 | 8 | 9 | 10 | 11 | 12 | 13 |
| --- | --- | --- | --- | --- | --- | --- | --- | --- | --- | --- | --- | --- | --- |
| 1 | 1 | .745  (*P*<.001) | -.321  (*P*=.007) | .396  (*P*<.001) | .384  (*P*=.001) | -.288  (*P*=.016) | .571  (*P*<.001) | .505  (*P*<.001) | -.144  (*P*=.234) | -.191  (*P*=.112) | -.322  (*P*=.007) | .247  (*P*=.039) | -.144  (*P*=.234) |
| 2 |  | 1 | -.478  (*P*<.001) | .517  (*P*<.001) | .507  (*P*<.001) | -.405  (*P*<.001) | .614  (*P*<.001) | .530  (*P*<.001) | -.096  (*P*=.430) | -.156  (*P*=.198) | -.261  (*P*=.029) | .217  (*P*=.071) | -.178  (*P*=.140) |
| 3 |  |  | 1 | -.284  (*P*=.017) | -.103  (*P*=.397) | .791  (*P*<.001) | -.281  (*P*=.019) | -.444  (*P*<.001) | .497  (*P*<.001) | .586  (*P*<.001) | .609  (*P*<.001) | -.233  (*P*=.052) | .456  (*P*<.001) |
| 4 |  |  |  | 1 | .402  (*P*<.001) | -.290  (*P*=.015) | .594  (*P*<.001) | .365  (*P*=.002) | -.166  (*P*=.169) | -.060  (*P*=.623) | -.136  (*P*=.261) | .172  (*P*=.156) | .005  (*P*=.968) |
| 5 |  |  |  |  | 1 | -.103  (*P*=.398) | .654  (*P*<.001) | .159  (*P*=.190) | .241  (*P*=.045) | .255  (*P*=.033) | -.068  (*P*=.576) | .006  (*P*=.959) | .231  (*P*=.054) |
| 6 |  |  |  |  |  | 1 | -.277  (*P*=.020) | -.427  (*P*<.001) | .562  (*P*<.001) | .625  (*P*<.001) | .642  (*P*<.001) | -.083  (*P*=.493) | .515  (*P*<.001) |
| 7 |  |  |  |  |  |  | 1 | .467  (*P*<.001) | .052  (*P*=.670) | .009  (*P*=.941) | -.168  (*P*=.164) | .154  (*P*=.202) | .043  (*P*=.723) |
| 8 |  |  |  |  |  |  |  | 1 | -.064  (*P*=.598) | -.314  (*P*=.008) | -.234  (*P*=.051) | .422  (*P*<.001) | -.182  (*P*=.131) |
| 9 |  |  |  |  |  |  |  |  | 1 | .757  (*P*<.001) | .508  (*P*<.001) | -.053  (*P*=.661) | .556  (*P*<.001) |
| 10 |  |  |  |  |  |  |  |  |  | 1 | .485  (*P*<.001) | -.038  (*P*=.752) | .671  (*P*<.001) |
| 11 |  |  |  |  |  |  |  |  |  |  | 1 | -.242  (*P*=.044) | .368  (*P*=.002) |
| 12 |  |  |  |  |  |  |  |  |  |  |  | 1 | .119  (*P*=.326) |
| 13 |  |  |  |  |  |  |  |  |  |  |  |  | 1 |

1=Intention; 2=Attitude(HIS); 3=Fear(HIS); 4=Perceived Norms; 5=Perceived Response Efficacy; 6=Abilities(HIS); 7=Perceived Cost/Barriers(HIS); 8=Perceived Behavioral Control; 9=Perceived Vulnerability; 10=Perceived Severity; 11=Nonadaptive Rewards; 12=Attitude (Disease); 13=Fear (Disease)

Table S10. Path coefficients according to the hypotheses, n = 496, location = Germany, time frame = April – May 2021

|  | Cataract physician – Cataract AI-supported physician | Cataract physician – Cataract AI | Cataract physician – Arthrosis physician | Cataract physician – Arthrosis AI-supported physician | Cataract physician – Arthrosis AI | Cataract AI-supported physician – Cataract AI | Cataract AI-supported physician – Arthrosis physician | Cataract AI-supported physician – Arthrosis AI-supported physician | Cataract AI-supported physician – Arthrosis AI | Cataract AI – Arthrosis physician | Cataract AI – Arthrosis AI-supported physician | Cataract AI – Arthrosis AI | Arthrosis physician – Arthrosis AI-supported physician | Arthrosis physician – Arthrosis AI | Arthrosis AI-supported physician – Arthrosis AI | Diabetes physician – Diabetes AI-supported physician | Diabetes physician – Diabetes AI | Diabetes AI-supported physician – Diabetes AI |
| --- | --- | --- | --- | --- | --- | --- | --- | --- | --- | --- | --- | --- | --- | --- | --- | --- | --- | --- |
| H1a | .427  (*P*<.001)  (f^2^=0.216)  .271  (*P*=.023)  (f^2^=0.083)  [-.247,.247]  .156  (*P*=.161) | .427  (*P*<.001)  (f^2^=0.216)  .161  (*P*=.103)  (f^2^=0.027)  [-.265,.260]  .266  (*P*=.046) | .427  (*P*<.001)  (f^2^=0.216)  .232  (*P*=.004)  (f^2^=0.067)  [-.205,.203]  .195  (*P*=.059) | .427  (*P*<.001)  (f^2^=0.216)  .571  (*P*<.001)  (f^2^=0.614)  [-.181,.175]  -.144  (*P*=.100) | .427  (*P*<.001)  (f^2^=0.216)  .540  (*P*<.001)  (f^2^=0.389)  [-.201,.203]  -.112  (*P*=.176) | .271  (*P*=.023)  (f^2^=0.083)  .161  (*P*=.103)  (f^2^=0.027)  [-.301,.313]  .110  (*P*=.272) | .271  (*P*=.023)  (f^2^=0.083)  .232  (*P*=.004)  (f^2^=0.067)  [-.247,.241]  .039  (*P*=.389) | .271  (*P*=.023)  (f^2^=0.083)  .571  (*P*<.001)  (f^2^=0.614)  [-.244,.242]  -.300  (*P*=.019) | .271  (*P*=.023)  (f^2^=0.083)  .540  (*P*<.001)  (f^2^=0.389)  [-.247,.247]  -.268  (*P*=.036) | .161  (*P*=.103)  (f^2^=0.027)  .232  (*P*=.004)  (f^2^=0.067)  [-.254,.252]  -.071  (*P*=.332) | .161  (*P*=.103)  (f^2^=0.027)  .571  (*P*<.001)  (f^2^=0.614)  [-.261,.251]  -.410  (*P*=.006) | .161  (*P*=.103)  (f^2^=0.027)  .540  (*P*<.001)  (f^2^=0.389)  [-.266,.254]  -.378  (*P*=.009) | .232  (*P*=.004)  (f^2^=0.067)  .571  (*P*<.001)  (f^2^=0.614)  [-.191,.204]  -.339  (*P*=.002) | .232  (*P*=.004)  (f^2^=0.067)  .540  (*P*<.001)  (f^2^=0.389)  [-.209,.207]  -.308  (*P*=.007) | .571  (*P*<.001)  (f^2^=0.614)  .540  (*P*<.001)  (f^2^=0.389)  [-.201,.200]  .032  (*P*=.399) | .714  (*P*<.001)  (f^2^=0.815)  .809  (*P*<.001)  (f^2^=0.655)  [-.260,.251]  -.095  (*P*=.279) | .714  (*P*<.001)  (f^2^=0.815)  .585  (*P*<.001)  (f^2^=0.444)  [-.164,.157]  .129  (*P*=.200) | .809  (*P*<.001)  (f^2^=0.655)  .585  (*P*<.001)  (f^2^=0.444)  [-.289,.288]  .224  (*P*=.102) |
| H1b | .031  (*P*=.342)  (f^2^=0.003)  -.042  (*P*=.340)  (f^2^=0.003)  [-.179,.174]  .073  (*P*=.246) | .031  (*P*=.342)  (f^2^=0.003)  .047  (*P*=.307)  (f^2^=0.006)  [-.173,.171]  -.016  (*P*=.023) | .031  (*P*=.342)  (f^2^=0.003)  -.124  (*P*=.015)  (f^2^=0.047)  [-.140,.136]  .154  (*P*=.033) | .031  (*P*=.342)  (f^2^=0.003)  -.052  (*P*=.163)  (f^2^=0.013)  [-.133,.132]  .083  (*P*=.151) | .031  (*P*=.342)  (f^2^=0.003)  -.113  (*P*=.109)  (f^2^=0.025)  [-.174,.179]  .143  (*P*=.090) | -.042  (*P*=.340)  (f^2^=0.003)  .047  (*P*=.307)  (f^2^=0.006)  [-.197,.194]  -.089  (*P*=.233) | -.042  (*P*=.340)  (f^2^=0.003)  -.124  (*P*=.015)  (f^2^=0.047)  [-.166,.164]  .081  (*P*=.359) | -.042  (*P*=.340)  (f^2^=0.003)  -.052  (*P*=.163)  (f^2^=0.013)  [-.175,.175]  .010  (*P*=.465) | -.042  (*P*=.340)  (f^2^=0.003)  -.113  (*P*=.109)  (f^2^=0.025)  [-.207,.205]  .071  (*P*=.283) | .047  (*P*=.307)  (f^2^=0.006)  -.124  (*P*=.015)  (f^2^=0.047)  [-.155,.159]  .170  (*P*=.038) | .047  (*P*=.307)  (f^2^=0.006)  -.052  (*P*=.163)  (f^2^=0.013)  [-.166,.166]  .099  (*P*=.170) | .047  (*P*=.307)  (f^2^=0.006)  -.113  (*P*=.109)  (f^2^=0.025)  [-.212,.210]  .159  (*P*=.110) | -.124  (*P*=.015)  (f^2^=0.047)  -.052  (*P*=.163)  (f^2^=0.013)  [-.129,.129]  -.071  (*P*=.186) | -.124  (*P*=.015)  (f^2^=0.047)  -.113  (*P*=.109)  (f^2^=0.025)  [-.155,.154]  -.011  (*P*=.465) | -.052  (*P*=.163)  (f^2^=0.013)  -.113  (*P*=.109)  (f^2^=0.025)  [-.160,.167]  .061  (*P*=.280) | .240  (*P*=.024)  (f^2^=0.080)  .068  (*P*=0.347)  (f^2^=0.005)  [-.315,.319]  .173  (*P*=.195) | .240  (*P*=.024)  (f^2^=0.080)  -.131  (*P*=.130)  (f^2^=0.025)  [-.304,.299]  .371  (*P*=.004) | .068  (*P*=0.347)  (f^2^=0.005)  -.131  (*P*=.130)  (f^2^=0.025)  [-.282,.279]  .199  (*P*=.004) |
| H2a | not assigned | not assigned | not assigned | not assigned | not assigned | not assigned | not assigned | not assigned | not assigned | not assigned | not assigned | not assigned | not assigned | not assigned | not assigned | .006  (*P*=.478)  (f^2^=0.000)  -.156  (*P*=0.146)  (f^2^=0.030)  [-.292,.276]  .162  (*P*=.170) | .006  (*P*=.478)  (f^2^=0.000)  .063  (*P*=.297)  (f^2^=0.005)  [-.269,.264]  -.056  (*P*=.367) | -.156  (*P*=0.146)  (f^2^=0.030)  .063  (*P*=.297)  (f^2^=0.005)  [-.322,.330]  -.219  (*P*=.137) |
| H2b | not assigned | not assigned | not assigned | not assigned | not assigned | not assigned | not assigned | not assigned | not assigned | not assigned | not assigned | not assigned | not assigned | not assigned | not assigned | -.038  (*P*=.379)  (f^2^=0.002)  -.001  (*P*=0.497)  (f^2^=0.000)  [-.282,.265]  -.036  (*P*=.420) | -.038  (*P*=.379)  (f^2^=0.002)  -.226  (*P*=.027)  (f^2^=0.075)  [-.286,.291]  .189  (*P*=.148) | -.001  (*P*=0.497)  (f^2^=0.000)  -.226  (*P*=.027)  (f^2^=0.075)  [-.303,.314]  .225  (*P*=.123) |
| H3 | .285  (*P*<.001)  (f^2^=0.142)  -.062  (*P*=.348)  (f^2^=0.004)  [-.309,.311]  .347  (*P*=.031) | .285  (*P*<.001)  (f^2^=0.142)  .311  (*P*=.018)  (f^2^=0.104)  [-.255,.247]  -.026  (*P*=.433) | .285  (*P*<.001)  (f^2^=0.142)  .051  (*P*=.269)  (f^2^=0.002)  [-.193,.195]  .234  (*P*=.024) | .285  (*P*<.001)  (f^2^=0.142)  .081  (*P*=.181)  (f^2^=0.006)  [-.202,.202]  .204  (*P*=.048) | .285  (*P*<.001)  (f^2^=0.142)  -.155  (*P*=.044)  (f^2^=0.027)  [-.292,.293]  .440  (*P*=.002) | -.062  (*P*=.348)  (f^2^=0.004)  .311  (*P*=.018)  (f^2^=0.104)  [-.401,.386]  -.373  (*P*=.065) | -.062  (*P*=.348)  (f^2^=0.004)  .051  (*P*=.269)  (f^2^=0.002)  [-.292,.287]  -.113  (*P*=.261) | -.062  (*P*=.348)  (f^2^=0.004)  .081  (*P*=.181)  (f^2^=0.006)  [-.354,.361]  -.144  (*P*=.282) | -.062  (*P*=.348)  (f^2^=0.004)  -.155  (*P*=.044)  (f^2^=0.027)  [-.270,.269]  .093  (*P*=.285) | .311  (*P*=.018)  (f^2^=0.104)  .051  (*P*=.269)  (f^2^=0.002)  [-.272,.273]  .260  (*P*=.060) | .311  (*P*=.018)  (f^2^=0.104)  .081  (*P*=.181)  (f^2^=0.006)  [-.288,.287]  .230  (*P*=.097) | .311  (*P*=.018)  (f^2^=0.104)  -.155  (*P*=.044)  (f^2^=0.027)  [-.297,.298]  .466  (*P*=.003) | .051  (*P*=.269)  (f^2^=0.002)  .081  (*P*=.181)  (f^2^=0.006)  [-.199,.196]  -.030  (*P*=.398) | .051  (*P*=.269)  (f^2^=0.002)  -.155  (*P*=.044)  (f^2^=0.027)  [-.207,.203]  .206  (*P*=.048) | .081  (*P*=.181)  (f^2^=0.006)  -.155  (*P*=.044)  (f^2^=0.027)  [-.215,.220]  .236  (*P*=.037) | .457  (*P*=.001)  (f^2^=0.235)  .456  (*P*=0.003)  (f^2^=0.270)  [-.349,.371]  .001  (*P*=.498) | .457  (*P*=.001)  (f^2^=0.235)  .081  (*P*=.308)  (f^2^=0.005)  [-.377,.280]  .376  (*P*=.052) | .456  (*P*=0.003)  (f^2^=0.270)  .081  (*P*=.308)  (f^2^=0.005)  [-.414,.403]  .375  (*P*=.004) |
| H4 | -.205  (*P*=.069)  (f^2^=0.089)  -.138  (*P*=.121)  (f^2^=0.035)  [-.313,.311]  -.067  (*P*=.358) | -.205  (*P*=.069)  (f^2^=0.089)  .013  (*P*=.421)  (f^2^=0.000)  [-.247,.236]  -.218  (*P*=.077) | -.205  (*P*=.069)  (f^2^=0.089)  -.010  (*P*=.440)  (f^2^=0.000)  [-.185,.199]  -.196  (*P*=.041) | -.205  (*P*=.069)  (f^2^=0.089)  -.136  (*P*=.022)  (f^2^=0.076)  [-.241,.233]  -.069  (*P*=.331) | -.205  (*P*=.069)  (f^2^=0.089)  -.102  (*P*=.194)  (f^2^=0.020)  [-.272,.264]  -.104  (*P*=.280) | -.138  (*P*=.121)  (f^2^=0.035)  .013  (*P*=.421)  (f^2^=0.000)  [-.226,.228]  -.151  (*P*=.150) | -.138  (*P*=.121)  (f^2^=0.035)  -.010  (*P*=.440)  (f^2^=0.000)  [-.236,.227]  -.129  (*P*=.198) | -.138  (*P*=.121)  (f^2^=0.035)  -.136  (*P*=.022)  (f^2^=0.076)  [-.264,.253]  -.002  (*P*=.486) | -.138  (*P*=.121)  (f^2^=0.035)  -.102  (*P*=.194)  (f^2^=0.020)  [-.273,.273]  -.037  (*P*=.420) | .013  (*P*=.421)  (f^2^=0.000)  -.010  (*P*=.440)  (f^2^=0.000)  [-.154,.155]  .023  (*P*=.403) | .013  (*P*=.421)  (f^2^=0.000)  -.136  (*P*=.022)  (f^2^=0.076)  [-.144,.141]  .149  (*P*=.040) | .013  (*P*=.421)  (f^2^=0.000)  -.102  (*P*=.194)  (f^2^=0.020)  [-.233,.230]  .115  (*P*=.267) | -.010  (*P*=.440)  (f^2^=0.000)  -.136  (*P*=.022)  (f^2^=0.076)  [-.150,.148]  .126  (*P*=.083) | -.010  (*P*=.440)  (f^2^=0.000)  -.102  (*P*=.194)  (f^2^=0.020)  [-.218,.217]  .092  (*P*=.264) | -.136  (*P*=.022)  (f^2^=0.076)  -.102  (*P*=.194)  (f^2^=0.020)  [-.279,.256]  -.034  (*P*=.446) | -.184  (*P*=.027)  (f^2^=0.056)  -.112  (*P*=0.234)  (f^2^=0.021)  [-.263,.258]  -.072  (*P*=.337) | -.184  (*P*=.027)  (f^2^=0.056)  .031  (*P*=.389)  (f^2^=0.002)  [-.205,.202]  -.215  (*P*=.039) | -.112  (*P*=0.234)  (f^2^=0.021)  .031  (*P*=.389)  (f^2^=0.002)  [-.266,.268]  -.143  (*P*=.200) |
| H5a | -.390  (*P*=.005)  (f^2^=0.110)  -.152  (*P*=.101)  (f^2^=0.020)  [-.334,.340]  -.238  (*P*=.125) | -.390  (*P*=.005)  (f^2^=0.110)  .021  (*P*=.429)  (f^2^=0.001)  [-.372,.332]  -.411  (*P*=.016) | -.390  (*P*=.005)  (f^2^=0.110)  -.124  (*P*=.113)  (f^2^=0.014)  [-.320,.335]  -.266  (*P*=.088) | -.390  (*P*=.005)  (f^2^=0.110)  -.328  (*P*=.012)  (f^2^=0.087)  [-.386,.400]  -.062  (*P*=.389) | -.390  (*P*=.005)  (f^2^=0.110)  -.143  (*P*=.115)  (f^2^=0.024)  [-.346,.351]  -.247  (*P*=.117) | -.152  (*P*=.101)  (f^2^=0.020)  .021  (*P*=.429)  (f^2^=0.001)  [-.233,.243]  -.173  (*P*=.115) | -.152  (*P*=.101)  (f^2^=0.020)  -.124  (*P*=.113)  (f^2^=0.014)  [-.240,.246]  -.027  (*P*=.425) | -.152  (*P*=.101)  (f^2^=0.020)  -.328  (*P*=.012)  (f^2^=0.087)  [-.317,.319]  .176  (*P*=.195) | -.152  (*P*=.101)  (f^2^=0.020)  -.143  (*P*=.115)  (f^2^=0.024)  [-.273,.281]  -.009  (*P*=.474) | .021  (*P*=.429)  (f^2^=0.001)  -.124  (*P*=.113)  (f^2^=0.014)  [-.233,.233]  .145  (*P*=.331) | .021  (*P*=.429)  (f^2^=0.001)  -.328  (*P*=.012)  (f^2^=0.087)  [-.287,.285]  .349  (*P*=.021) | .021  (*P*=.429)  (f^2^=0.001)  -.143  (*P*=.115)  (f^2^=0.024)  [-.262,.264]  .164  (*P*=.158) | -.124  (*P*=.113)  (f^2^=0.014)  -.328  (*P*=.012)  (f^2^=0.087)  [-.282,.274]  .204  (*P*=.107) | -.124  (*P*=.113)  (f^2^=0.014)  -.143  (*P*=.115)  (f^2^=0.024)  [-.261,.254]  .019  (*P*=.450) | -.328  (*P*=.012)  (f^2^=0.087)  -.143  (*P*=.115)  (f^2^=0.024)  [-.310,.322]  -.185  (*P*=.171) | -.176  (*P*=.105)  (f^2^=0.029)  -.345  (*P*=0.006)  (f^2^=0.069)  [-.293,.286]  .168  (*P*=.169) | -.176  (*P*=.105)  (f^2^=0.029)  -.287  (*P*=.005)  (f^2^=0.091)  [-.311,.303]  .111  (*P*=.263) | -.345  (*P*=0.006)  (f^2^=0.069)  -.287  (*P*=.005)  (f^2^=0.091)  [-.291,.279]  -.057  (*P*=.374) |
| H5b | .476  (*P*<.001)  (f^2^=0.470)  .031  (*P*=.394)  (f^2^=0.001)  [-.265,.266]  .445  (*P*=.003) | .476  (*P*<.001)  (f^2^=0.470)  .272  (*P*<.001)  (f^2^=0.148)  [-.180,.181]  .204  (*P*=.028) | .476  (*P*<.001)  (f^2^=0.470)  .342  (*P*=.001)  (f^2^=0.171)  [-.281,.270]  .134  (*P*=.218) | .476  (*P*<.001)  (f^2^=0.470)  .319  (*P*=.002)  (f^2^=0.135)  [-.289,.269]  .158  (*P*=.180) | .476  (*P*<.001)  (f^2^=0.470)  .408  (*P*<.001)  (f^2^=0.347)  [-.211,.207]  .068  (*P*=.307) | .031  (*P*=.394)  (f^2^=0.001)  .272  (*P*<.001)  (f^2^=0.148)  [-.189,.183]  -.241  (*P*=.015) | .031  (*P*=.394)  (f^2^=0.001)  .342  (*P*=.001)  (f^2^=0.171)  [-.275,.271]  -.312  (*P*=.030) | .031  (*P*=.394)  (f^2^=0.001)  .319  (*P*=.002)  (f^2^=0.135)  [-.247,.243]  -.288  (*P*=.029) | .031  (*P*=.394)  (f^2^=0.001)  .408  (*P*<.001)  (f^2^=0.347)  [-.215,.218]  -.377  (*P*=.001) | .272  (*P*<.001)  (f^2^=0.148)  .342  (*P*=.001)  (f^2^=0.171)  [-.211,.214]  -.070  (*P*=.306) | .272  (*P*<.001)  (f^2^=0.148)  .319  (*P*=.002)  (f^2^=0.135)  [-.200,.202]  -.047  (*P*=.348) | .272  (*P*<.001)  (f^2^=0.148)  .408  (*P*<.001)  (f^2^=0.347)  [-.173,.178]  -.136  (*P*=.102) | .342  (*P*=.001)  (f^2^=0.171)  .319  (*P*=.002)  (f^2^=0.135)  [-.260,.255]  .024  (*P*=.453) | .342  (*P*=.001)  (f^2^=0.171)  .408  (*P*<.001)  (f^2^=0.347)  [-.211,.221]  -.066  (*P*=.299) | .319  (*P*=.002)  (f^2^=0.135)  .408  (*P*<.001)  (f^2^=0.347)  [-.207,.204]  -.089  (*P*=.245) | .497  (*P*<.001)  (f^2^=0.529)  .716  (*P*<.001)  (f^2^=0.549)  [-.294,.293]  -.219  (*P*=.109) | .497  (*P*<.001)  (f^2^=0.529)  .466  (*P*<.001)  (f^2^=0.287)  [-.251,.250]  .031  (*P*=.414) | .716  (*P*<.001)  (f^2^=0.549)  .466  (*P*<.001)  (f^2^=0.287)  [-.325,.313]  .249  (*P*=.097) |
| H5c | .719  (*P*<.001)  (f^2^=0.784)  .186  (*P*=.049)  (f^2^=0.044)  [-.326,.334]  .533  (*P*=.002) | .719  (*P*<.001)  (f^2^=0.784)  .102  (*P*=.111)  (f^2^=0.020)  [-.305,.305]  .617  (*P*<.001) | .719  (*P*<.001)  (f^2^=0.784)  .010  (*P*=.451)  (f^2^=0.000)  [-.350,.336]  .709  (*P*<.001) | .719  (*P*<.001)  (f^2^=0.784)  .217  (*P*=.007)  (f^2^=0.089)  [-.325,.327]  .502  (*P*=.003) | .719  (*P*<.001)  (f^2^=0.784)  .184  (*P*=.068)  (f^2^=0.040)  [-.353,.357]  .536  (*P*=.003) | .186  (*P*=.049)  (f^2^=0.044)  .102  (*P*=.111)  (f^2^=0.020)  [-.232,.226]  .084  (*P*=.263) | .186  (*P*=.049)  (f^2^=0.044)  .010  (*P*=.451)  (f^2^=0.000)  [-.222,.228]  .176  (*P*=.101) | .186  (*P*=.049)  (f^2^=0.044)  .217  (*P*=.007)  (f^2^=0.089)  [-.250,.254]  -.031  (*P*=.418) | .186  (*P*=.049)  (f^2^=0.044)  .184  (*P*=.068)  (f^2^=0.040)  [-.237,.240]  .003  (*P*=.496) | .102  (*P*=.111)  (f^2^=0.020)  .010  (*P*=.451)  (f^2^=0.000)  [-.184,.182]  .092  (*P*=.208) | .102  (*P*=.111)  (f^2^=0.020)  .217  (*P*=.007)  (f^2^=0.089)  [-.218,.207]  -.115  (*P*=.187) | .102  (*P*=.111)  (f^2^=0.020)  .184  (*P*=.068)  (f^2^=0.040)  [-.205,.213]  -.082  (*P*=.109) | .010  (*P*=.451)  (f^2^=0.000)  .217  (*P*=.007)  (f^2^=0.089)  [-.197,.203]  -.207  (*P*=.041) | .010  (*P*=.451)  (f^2^=0.000)  .184  (*P*=.068)  (f^2^=0.040)  [-.214,.227]  -.174  (*P*=.095) | .217  (*P*=.007)  (f^2^=0.089)  .184  (*P*=.068)  (f^2^=0.040)  [-.258,.268]  .033  (*P*=.423) | -.094  (*P*=.189)  (f^2^=0.010)  .271  (*P*=0.102)  (f^2^=0.043)  [-.355,.350]  -.365  (*P*=.046) | -.094  (*P*=.189)  (f^2^=0.010)  .285  (*P*=.031)  (f^2^=0.098)  [-.346,.364]  -.379  (*P*=.032) | .271  (*P*=0.102)  (f^2^=0.043)  .285  (*P*=.031)  (f^2^=0.098)  [-.398,.405]  -.014  (*P*=.466) |
| H6 | -.121  (*P*=.059)  (f^2^=0.036)  -.337  (*P*<.001)  (f^2^=0.238)  [-.199,.195]  .216  (*P*=.035) | -.121  (*P*=.059)  (f^2^=0.036)  -.392  (*P*<.001)  (f^2^=0.317)  [-.154,.159]  .271  (*P*=.001) | -.121  (*P*=.059)  (f^2^=0.036)  -.143  (*P*=.066)  (f^2^=0.034)  [-.191,.196]  .022  (*P*=.434) | -.121  (*P*=.059)  (f^2^=0.036)  -.245  (*P*=.001)  (f^2^=0.122)  [-.158,.158]  .124  (*P*=.099) | -.121  (*P*=.059)  (f^2^=0.036)  -.189  (*P*=.056)  (f^2^=0.079)  [-.223,.226]  .068  (*P*=.322) | -.337  (*P*<.001)  (f^2^=0.238)  -.392  (*P*<.001)  (f^2^=0.317)  [-.178,.171]  .054  (*P*=.301) | -.337  (*P*<.001)  (f^2^=0.238)  -.143  (*P*=.066)  (f^2^=0.034)  [-.225,.224]  -.194  (*P*=.074) | -.337  (*P*<.001)  (f^2^=0.238)  -.245  (*P*=.001)  (f^2^=0.122)  [-.197,.202]  -.092  (*P*=.229) | -.337  (*P*<.001)  (f^2^=0.238)  -.189  (*P*=.056)  (f^2^=0.079)  [-.275,.283]  -.148  (*P*=.202) | -.392  (*P*<.001)  (f^2^=0.317)  -.143  (*P*=.066)  (f^2^=0.034)  [-.192,.206]  -.249  (*P*=.015) | -.392  (*P*<.001)  (f^2^=0.317)  -.245  (*P*=.001)  (f^2^=0.122)  [-.163,.159]  -.147  (*P*=.071) | -.392  (*P*<.001)  (f^2^=0.317)  -.189  (*P*=.056)  (f^2^=0.079)  [-.252,.253]  -.202  (*P*=.100) | -.143  (*P*=.066)  (f^2^=0.034)  -.245  (*P*=.001)  (f^2^=0.122)  [-.205,.196]  .102  (*P*=.200) | -.143  (*P*=.066)  (f^2^=0.034)  -.189  (*P*=.056)  (f^2^=0.079)  [-.258,.257]  .047  (*P*=.385) | -.245  (*P*=.001)  (f^2^=0.122)  -.189  (*P*=.056)  (f^2^=0.079)  [-.253,.267]  -.056  (*P*=.384) | -.191  (*P*=.025)  (f^2^=0.051)  -.184  (*P*=0.069)  (f^2^=0.045)  [-.268,.263]  -.007  (*P*=.482) | -.191  (*P*=.025)  (f^2^=0.051)  -.272  (*P*=.081)  (f^2^=0.047)  [-.346,.355]  .081  (*P*=.354) | -.184  (*P*=0.069)  (f^2^=0.045)  -.272  (*P*=.081)  (f^2^=0.047)  [-.408,.398]  .088  (*P*=.367) |
| H7 | .285  (*P*=.069)  (f^2^=0.087)  .447  (*P*=.002)  (f^2^=0.221)  [-.327,.321]  -.163  (*P*=.056) | .285  (*P*=.069)  (f^2^=0.087)  .624  (*P*<.001)  (f^2^=0.545)  [-.258,.260]  -.339  (*P*=.017) | .285  (*P*=.069)  (f^2^=0.087)  .701  (*P*<.001)  (f^2^=0.716)  [-.296,.296]  -.417  (*P*=.009) | .285  (*P*=.069)  (f^2^=0.087)  .703  (*P*<.001)  (f^2^=0.964)  [-.303,.306]  -.419  (*P*=.012) | .285  (*P*=.069)  (f^2^=0.087)  .413  (*P*=.035)  (f^2^=0.142)  [-.472,.470]  -.128  (*P*=.372) | .447  (*P*=.002)  (f^2^=0.221)  .624  (*P*<.001)  (f^2^=0.545)  [-.311,.311]  -.176  (*P*=.178) | .447  (*P*=.002)  (f^2^=0.221)  .701  (*P*<.001)  (f^2^=0.716)  [-.291,.285]  -.254  (*P*=.069) | .447  (*P*=.002)  (f^2^=0.221)  .703  (*P*<.001)  (f^2^=0.964)  [-.307,.300]  -.256  (*P*=.089) | .447  (*P*=.002)  (f^2^=0.221)  .413  (*P*=.035)  (f^2^=0.142)  [-.472,.447]  .035  (*P*=.457) | .624  (*P*<.001)  (f^2^=0.545)  .701  (*P*<.001)  (f^2^=0.716)  [-.217,.216]  -.077  (*P*=.274) | .624  (*P*<.001)  (f^2^=0.545)  .703  (*P*<.001)  (f^2^=0.964)  [-.231,.232]  -.079  (*P*=.292) | .624  (*P*<.001)  (f^2^=0.545)  .413  (*P*=.035)  (f^2^=0.142)  [-.415,.397]  .211  (*P*=.256) | .701  (*P*<.001)  (f^2^=0.716)  .703  (*P*<.001)  (f^2^=0.964)  [-.206,.216]  -.002  (*P*=.499) | .701  (*P*<.001)  (f^2^=0.716)  .413  (*P*=.035)  (f^2^=0.142)  [-.366,.378]  .289  (*P*=.132) | .703  (*P*<.001)  (f^2^=0.964)  .413  (*P*=.035)  (f^2^=0.142)  [-.447,.424]  .291  (*P*=.183) | .483  (*P*=.015)  (f^2^=0.187)  .431  (*P*=0.005)  (f^2^=0.166)  [-.398,.374]  .052  (*P*=.409) | .483  (*P*=.015)  (f^2^=0.187)  .614  (*P*<.001)  (f^2^=0.527)  [-.322,.310]  -.131  (*P*=.254) | .431  (*P*=0.005)  (f^2^=0.166)  .614  (*P*<.001)  (f^2^=0.527)  [-.319,.303]  -.183  (*P*=.168) |
| H8a | -.170  (*P*.=118)  (f^2^=0.030)  .179  (*P*.=132)  (f^2^=0.032)  [-.354,.352]  -.349  (*P*=.054) | -.170  (*P*.=118)  (f^2^=0.030)  .215  (*P*=.048)  (f^2^=0.049)  [-.312,.315]  -.385  (*P*=.021) | -.170  (*P*.=118)  (f^2^=0.030)  .167  (*P*=.065)  (f^2^=0.029)  [-.261,.274]  -.337  (*P*=.031) | -.170  (*P*.=118)  (f^2^=0.030)  .143  (*P*=.133)  (f^2^=0.020)  [-.295,.290]  -.314  (*P*=.041) | -.170  (*P*.=118)  (f^2^=0.030)  .110  (*P*=.185)  (f^2^=0.013)  [-.291,.293]  -.280  (*P*=.056) | .179  (*P*.=132)  (f^2^=0.032)  .215  (*P*=.048)  (f^2^=0.049)  [-.323,.323]  -.036  (*P*=.428) | .179  (*P*.=132)  (f^2^=0.032)  .167  (*P*=.065)  (f^2^=0.029)  [-.310,.289]  .011  (*P*=.331) | .179  (*P*.=132)  (f^2^=0.032)  .143  (*P*=.133)  (f^2^=0.020)  [-.332,.333]  .035  (*P*=.424) | .179  (*P*.=132)  (f^2^=0.032)  .110  (*P*=.185)  (f^2^=0.013)  [-.313,.329]  .069  (*P*=.367) | .215  (*P*=.048)  (f^2^=0.049)  .167  (*P*=.065)  (f^2^=0.029)  [-.297,.288]  .048  (*P*=.331) | .215  (*P*=.048)  (f^2^=0.049)  .143  (*P*=.133)  (f^2^=0.020)  [-.315,.310]  .071  (*P*=.352) | .215  (*P*=.048)  (f^2^=0.049)  .110  (*P*=.185)  (f^2^=0.013)  [-.292,.293]  .105  (*P*=.292) | .167  (*P*=.065)  (f^2^=0.029)  .143  (*P*=.133)  (f^2^=0.020)  [-.280,.282]  .024  (*P*=.453) | .167  (*P*=.065)  (f^2^=0.029)  .110  (*P*=.185)  (f^2^=0.013)  [-.277,.266]  .057  (*P*=.368) | .143  (*P*=.133)  (f^2^=0.020)  .110  (*P*=.185)  (f^2^=0.013)  [-.294,.283]  .034  (*P*=.225) | .322  (*P*=.006)  (f^2^=0.136)  .218  (*P*=.052)  (f^2^=0.060)  [-.318,.327]  .103  (*P*=.307) | .322  (*P*=.006)  (f^2^=0.136)  -.146  (*P*=.186)  (f^2^=0.026)  [-.386,.367]  .467  (*P*=.018) | .218  (*P*=.052)  (f^2^=0.060)  -.146  (*P*=.186)  (f^2^=0.026)  [-.366,.379]  .364  (*P*=.057) |
| H8b | .132  (*P*=.139)  (f^2^=0.021)  .042  (*P*.=369)  (f^2^=0.002)  [-.298,.295]  .091  (*P*=.306) | .132  (*P*=.139)  (f^2^=0.021)  -.021  (*P*=.434)  (f^2^=0.024)  [-.296,.299]  .154  (*P*=.208) | .132  (*P*=.139)  (f^2^=0.021)  .289  (*P*=.001)  (f^2^=0.104)  [-.252,.254]  -.156  (*P*=.151) | .132  (*P*=.139)  (f^2^=0.021)  .241  (*P*=.012)  (f^2^=0.107)  [-.257,.256]  -.109  (*P*=.241) | .132  (*P*=.139)  (f^2^=0.021)  -.018  (*P*=.427)  (f^2^=0.000)  [-.256,.258]  .151  (*P*=.158) | .042  (*P*.=369)  (f^2^=0.002)  -.021  (*P*=.434)  (f^2^=0.024)  [-.290,.290]  .063  (*P*=.350) | .042  (*P*.=369)  (f^2^=0.002)  .289  (*P*=.001)  (f^2^=0.104)  [-.270,.274]  -.247  (*P*=.065) | .042  (*P*.=369)  (f^2^=0.002)  .241  (*P*=.012)  (f^2^=0.107)  [-.280,.284]  -.200  (*P*=.123) | .042  (*P*.=369)  (f^2^=0.002)  -.018  (*P*=.427)  (f^2^=0.000)  [-.267,.274]  .060  (*P*=.348) | -.021  (*P*=.434)  (f^2^=0.024)  .289  (*P*=.001)  (f^2^=0.104)  [-.293,.292]  -.310  (*P*=.040) | -.021  (*P*=.434)  (f^2^=0.024)  .241  (*P*=.012)  (f^2^=0.107)  [-.328,.324]  -.263  (*P*=.093) | -.021  (*P*=.434)  (f^2^=0.024)  -.018  (*P*=.427)  (f^2^=0.000)  [-.290,.284]  -.003  (*P*=.491) | .289  (*P*=.001)  (f^2^=0.104)  .241  (*P*=.012)  (f^2^=0.107)  [-.247,.248]  .047  (*P*=.366) | .289  (*P*=.001)  (f^2^=0.104)  -.018  (*P*=.427)  (f^2^=0.000)  [-.253,.248]  .307  (*P*=.023) | .241  (*P*=.012)  (f^2^=0.107)  -.018  (*P*=.427)  (f^2^=0.000)  [-.258,.250]  .260  (*P*=.044) | .113  (*P*=.160)  (f^2^=0.023)  -.051  (*P*=.322)  (f^2^=0.005)  [-.253,.249]  .164  (*P*=.144) | .113  (*P*=.160)  (f^2^=0.023)  .037  (*P*=.358)  (f^2^=0.002)  [-.251,.263]  .076  (*P*=.320) | -.051  (*P*=.322)  (f^2^=0.005)  .037  (*P*=.358)  (f^2^=0.002)  [-.253,.248]  -.088  (*P*=.289) |
| H8c | .063  (*P*=.312)  (f^2^=0.008)  .224  (*P*=.049)  (f^2^=0.057)  [-.310,.310]  -.161  (*P*=.203) | .063  (*P*=.312)  (f^2^=0.008)  .108  (*P*=.173)  (f^2^=0.021)  [-.274,.278]  -.045  (*P*=.408) | .063  (*P*=.312)  (f^2^=0.008)  .049  (*P*=.233)  (f^2^=0.005)  [-.294,.254]  .014  (*P*=.481) | .063  (*P*=.312)  (f^2^=0.008)  .141  (*P*=.063)  (f^2^=0.049)  [-.267,.268]  -.078  (*P*=.330) | .063  (*P*=.312)  (f^2^=0.008)  .015  (*P*=.439)  (f^2^=0.000)  [-.290,.303]  .048  (*P*=.395) | .224  (*P*=.049)  (f^2^=0.057)  .108  (*P*=.173)  (f^2^=0.021)  [-.301,.293]  .116  (*P*=.265) | .224  (*P*=.049)  (f^2^=0.057)  .049  (*P*=.233)  (f^2^=0.005)  [-.220,.219]  .175  (*P*=.095) | .224  (*P*=.049)  (f^2^=0.057)  .141  (*P*=.063)  (f^2^=0.049)  [-.280,.274]  .083  (*P*=.310) | .224  (*P*=.049)  (f^2^=0.057)  .015  (*P*=.439)  (f^2^=0.000)  [-.254,.271]  .209  (*P*=.104) | .108  (*P*=.173)  (f^2^=0.021)  .049  (*P*=.233)  (f^2^=0.005)  [-.226,.229]  .059  (*P*=.339) | .108  (*P*=.173)  (f^2^=0.021)  .141  (*P*=.063)  (f^2^=0.049)  [-.253,.254]  -.033  (*P*=.424) | .108  (*P*=.173)  (f^2^=0.021)  .015  (*P*=.439)  (f^2^=0.000)  [-.231,.237]  .093  (*P*=.259) | .049  (*P*=.233)  (f^2^=0.005)  .141  (*P*=.063)  (f^2^=0.049)  [-.187,.180]  -.092  (*P*=.207) | .049  (*P*=.233)  (f^2^=0.005)  .015  (*P*=.439)  (f^2^=0.000)  [-.209,.210]  .034  (*P*=.402) | .141  (*P*=.063)  (f^2^=0.049)  .015  (*P*=.439)  (f^2^=0.000)  [-.222,.220]  .126  (*P*=.176) | .385  (*P*=.031)  (f^2^=0.204)  .113  (*P*=.236)  (f^2^=0.013)  [-.432,.428]  .272  (*P*=.164) | .385  (*P*=.031)  (f^2^=0.204)  -.044  (*P*=.356)  (f^2^=0.003)  [-.386,.367]  .429  (*P*=.044) | .113  (*P*=.236)  (f^2^=0.013)  -.044  (*P*=.356)  (f^2^=0.003)  [-.317,.306]  .157  (*P*=.206) |
| H8d | .379  (*P*=.001)  (f^2^=0.387)  .269  (*P*=.002)  (f^2^=0.112)  [-.235,.223]  .110  (*P*=.217) | .379  (*P*=.001)  (f^2^=0.387)  .261  (*P*=.002)  (f^2^=0.146)  [-.216,.228]  .119  (*P*=.197) | .379  (*P*=.001)  (f^2^=0.387)  .355  (*P*<.001)  (f^2^=0.210)  [-.222,.225]  .024  (*P*=.432) | .379  (*P*=.001)  (f^2^=0.387)  .313  (*P*<.001)  (f^2^=0.180)  [-.230,.238]  .066  (*P*=.335) | .379  (*P*=.001)  (f^2^=0.387)  .313  (*P*<.001)  (f^2^=0.247)  [-.290,.303]  .048  (*P*=.395) | .269  (*P*=.002)  (f^2^=0.112)  .261  (*P*=.002)  (f^2^=0.146)  [-.221,.224]  .008  (*P*=.472) | .269  (*P*=.002)  (f^2^=0.112)  .355  (*P*<.001)  (f^2^=0.210)  [-.198,.201]  -.086  (*P*=.239) | .269  (*P*=.002)  (f^2^=0.112)  .313  (*P*<.001)  (f^2^=0.180)  [-.184,.191]  -.044  (*P*=.355) | .269  (*P*=.002)  (f^2^=0.112)  .313  (*P*<.001)  (f^2^=0.247)  [-.191,.193]  -.044  (*P*=.346) | .261  (*P*=.002)  (f^2^=0.146)  .355  (*P*<.001)  (f^2^=0.210)  [-.202,.202]  -.094  (*P*=.229) | .261  (*P*=.002)  (f^2^=0.146)  .313  (*P*<.001)  (f^2^=0.180)  [-.211,.212]  -.052  (*P*=.334) | .261  (*P*=.002)  (f^2^=0.146)  .313  (*P*<.001)  (f^2^=0.247)  [-.197,.201]  -.052  (*P*=.341) | .355  (*P*<.001)  (f^2^=0.210)  .313  (*P*<.001)  (f^2^=0.180)  [-.187,.179]  .042  (*P*=.346) | .355  (*P*<.001)  (f^2^=0.210)  .313  (*P*<.001)  (f^2^=0.247)  [-.185,.184]  .042  (*P*=.346) | .313  (*P*<.001)  (f^2^=0.180)  .313  (*P*<.001)  (f^2^=0.247)  [-.179,.180]  .000  (*P*=.506) | .321  (*P*=.002)  (f^2^=0.265)  .015  (*P*=.462)  (f^2^=0.000)  [-.296,.285]  -.306  (*P*=.036) | .321  (*P*=.002)  (f^2^=0.265)  .226  (*P*=.026)  (f^2^=0.072)  [-.275,.271]  .095  (*P*=.288) | .015  (*P*=.462)  (f^2^=0.000)  .226  (*P*=.026)  (f^2^=0.072)  [-.314,.324]  -.210  (*P*=.140) |
| H8e | .329  (*P*=.006)  (f^2^=0.157)  .346  (*P*<.001)  (f^2^=0.146)  [-.260,.251]  -.018  (*P*=.459) | .329  (*P*=.006)  (f^2^=0.157)  .135  (*P*=.126)  (f^2^=0.021)  [-.274,.268]  .193  (*P*=.124) | .329  (*P*=.006)  (f^2^=0.157)  .388  (*P*<.001)  (f^2^=0.209)  [-.261,.274]  -.059  (*P*=.350) | .329  (*P*=.006)  (f^2^=0.157)  .414  (*P*<.001)  (f^2^=0.321)  [-.281,.288]  -.085  (*P*=.327) | .329  (*P*=.006)  (f^2^=0.157)  .136  (*P*=.118)  (f^2^=0.024)  [-.271,.275]  .193  (*P*=.125) | .346  (*P*<.001)  (f^2^=0.146)  .135  (*P*=.126)  (f^2^=0.021)  [-.261,.263]  .211  (*P*=.092) | .346  (*P*<.001)  (f^2^=0.146)  .388  (*P*<.001)  (f^2^=0.209)  [-.229,.230]  -.041  (*P*=.393) | .346  (*P*<.001)  (f^2^=0.146)  .414  (*P*<.001)  (f^2^=0.321)  [-.214,.213]  -.068  (*P*=.305) | .346  (*P*<.001)  (f^2^=0.146)  .136  (*P*=.118)  (f^2^=0.024)  [-.247,.242]  .210  (*P*=.076) | .135  (*P*=.126)  (f^2^=0.021)  .388  (*P*<.001)  (f^2^=0.209)  [-.259,.247]  -.252  (*P*=.055) | .135  (*P*=.126)  (f^2^=0.021)  .414  (*P*<.001)  (f^2^=0.321)  [-.244,.251]  -.279  (*P*=.030) | .135  (*P*=.126)  (f^2^=0.021)  .136  (*P*=.118)  (f^2^=0.024)  [-.272,.262]  -.001  (*P*=.498) | .388  (*P*<.001)  (f^2^=0.209)  .414  (*P*<.001)  (f^2^=0.321)  [-.250,.254]  -.027  (*P*=.437) | .388  (*P*<.001)  (f^2^=0.209)  .136  (*P*=.118)  (f^2^=0.024)  [-.250,.242]  .252  (*P*=.044) | .414  (*P*<.001)  (f^2^=0.321)  .136  (*P*=.118)  (f^2^=0.024)  [-.260,.256]  .278  (*P*=.035) | .386  (*P*<.001)  (f^2^=0.213)  .723  (*P*<.001)  (f^2^=1.252)  [-.237,.235]  -.337  (*P*=.009) | .386  (*P*<.001)  (f^2^=0.213)  .457  (*P*<.001)  (f^2^=0.291)  [-.280,.272]  -.072  (*P*=.352) | .723  (*P*<.001)  (f^2^=1.252)  .457  (*P*<.001)  (f^2^=0.291)  [-.244,.244]  .266  (*P*=.035) |
| H8f | .579  (*P*<.001)  (f^2^=0.590)  .114  (*P*=.221)  (f^2^=0.014)  [-.579,.114]  .465  (*P*=.004) | .579  (*P*<.001)  (f^2^=0.590)  -.167  (*P*=.045)  (f^2^=0.032)  [-.200,.199]  .746  (*P*<.001) | .579  (*P*<.001)  (f^2^=0.590)  .033  (*P*=.374)  (f^2^=0.001)  [-.285,.288]  .546  (*P*<.001) | .579  (*P*<.001)  (f^2^=0.590)  -.030  (*P*=.396)  (f^2^=0.001)  [-.274,.269]  .609  (*P*<.001) | .579  (*P*<.001)  (f^2^=0.590)  -.313  (*P*<.001)  (f^2^=0.112)  [-.228,.228]  .891  (*P*<.001) | .114  (*P*=.221)  (f^2^=0.014)  -.167  (*P*=.045)  (f^2^=0.032)  [-.271,.265]  .281  (*P*=.040) | .114  (*P*=.221)  (f^2^=0.014)  .033  (*P*=.374)  (f^2^=0.001)  [-.317,.320]  .081  (*P*=.359) | .114  (*P*=.221)  (f^2^=0.014)  -.030  (*P*=.396)  (f^2^=0.001)  [-.359,.349]  .144  (*P*=.310) | .114  (*P*=.221)  (f^2^=0.014)  -.313  (*P*<.001)  (f^2^=0.112)  [-.279,.277]  .426  (*P*=.002) | -.167  (*P*=.045)  (f^2^=0.032)  .033  (*P*=.374)  (f^2^=0.001)  [-.221,.220]  -.200  (*P*=.069) | -.167  (*P*=.045)  (f^2^=0.032)  -.030  (*P*=.396)  (f^2^=0.001)  [-.226,.225]  -.137  (*P*=.167) | -.167  (*P*=.045)  (f^2^=0.032)  -.313  (*P*<.001)  (f^2^=0.112)  [-.218,.218]  .146  (*P*=.137) | .033  (*P*=.374)  (f^2^=0.001)  -.030  (*P*=.396)  (f^2^=0.001)  [-.254,.249]  .063  (*P*=.338) | .033  (*P*=.374)  (f^2^=0.001)  -.313  (*P*<.001)  (f^2^=0.112)  [-.203,.201]  .345  (*P*=.001) | -.030  (*P*=.396)  (f^2^=0.001)  -.313  (*P*<.001)  (f^2^=0.112)  [-.224,.225]  .283  (*P*=.017) | -.116  (*P*=.245)  (f^2^=0.016)  .108  (*P*=.197)  (f^2^=0.016)  [-.348,.362]  -.224  (*P*=.153) | -.116  (*P*=.245)  (f^2^=0.016)  .180  (*P*=.109)  (f^2^=0.035)  [-.355,.354]  -.295  (*P*=.086) | .108  (*P*=.197)  (f^2^=0.016)  .180  (*P*=.109)  (f^2^=0.035)  [-.304,.310]  -.072  (*P*=.350) |
| H8g | .165  (*P*=.114)  (f^2^=0.021)  -.116  (*P*=.228)  (f^2^=0.009)  [-.343,.332]  .281  (*P*=.084) | .165  (*P*=.114)  (f^2^=0.021)  -.146  (*P*=.142)  (f^2^=0.024)  [-.326,.323]  .310  (*P*=.055) | .165  (*P*=.114)  (f^2^=0.021)  -.088  (*P*=.194)  (f^2^=0.007)  [-.313,.300]  .253  (*P*=.087) | .165  (*P*=.114)  (f^2^=0.021)  -.112  (*P*=.205)  (f^2^=0.010)  [-.366,.359]  .277  (*P*=.107) | .165  (*P*=.114)  (f^2^=0.021)  -.229  (*P*=.024)  (f^2^=0.063)  [-.347,.333]  .394  (*P*=.025) | -.116  (*P*=.228)  (f^2^=0.009)  -.146  (*P*=.142)  (f^2^=0.024)  [-.306,.306]  .030  (*P*=.430) | -.116  (*P*=.228)  (f^2^=0.009)  -.088  (*P*=.194)  (f^2^=0.007)  [-.264,.274]  -.028  (*P*=.437) | -.116  (*P*=.228)  (f^2^=0.009)  -.112  (*P*=.205)  (f^2^=0.010)  [-.334,.338]  -.004  (*P*=.502) | -.116  (*P*=.228)  (f^2^=0.009)  -.229  (*P*=.024)  (f^2^=0.063)  [-.305,.302]  .113  (*P*=.271) | -.146  (*P*=.142)  (f^2^=0.024)  -.088  (*P*=.194)  (f^2^=0.007)  [-.253,.259]  -.058  (*P*=.357) | -.146  (*P*=.142)  (f^2^=0.024)  -.112  (*P*=.205)  (f^2^=0.010)  [-.312,.300]  -.033  (*P*=.415) | -.146  (*P*=.142)  (f^2^=0.024)  -.229  (*P*=.024)  (f^2^=0.063)  [-.277,.277]  .083  (*P*=.109) | -.088  (*P*=.194)  (f^2^=0.007)  -.112  (*P*=.205)  (f^2^=0.010)  [-.276,.283]  .024  (*P*=.449) | -.088  (*P*=.194)  (f^2^=0.007)  -.229  (*P*=.024)  (f^2^=0.063)  [-.252,.255]  .141  (*P*=.185) | -.112  (*P*=.205)  (f^2^=0.010)  -.229  (*P*=.024)  (f^2^=0.063)  [-.294,.292]  .117  (*P*=.259) | .015  (*P*=.454)  (f^2^=0.000)  .268  (*P*=.052)  (f^2^=0.042)  [-.319,.334]  -.253  (*P*=.095) | .015  (*P*=.454)  (f^2^=0.000)  -.197  (*P*=.060)  (f^2^=0.043)  [-.322,.321]  .212  (*P*=.141) | .268  (*P*=.052)  (f^2^=0.042)  -.197  (*P*=.060)  (f^2^=0.043)  [-.367,.369]  .465  (*P*=.020) |
| H8h | not assigned | not assigned | not assigned | not assigned | not assigned | not assigned | not assigned | not assigned | not assigned | not assigned | not assigned | not assigned | not assigned | not assigned | not assigned | -.188  (*P*=.079)  (f^2^=0.045)  .026  (*P*=.442)  (f^2^=0.001)  [-.318,.326]  -.214  (*P*=.133) | -.188  (*P*=.079)  (f^2^=0.045)  -.185  (*P*=.081)  (f^2^=0.039)  [-.307,.300]  -.002  (*P*=.506) | .026  (*P*=.442)  (f^2^=0.001)  -.185  (*P*=.081)  (f^2^=0.039)  [-.319,.310]  .212  (*P*=.141) |
| H9a | .356  (*P*=.002)  (f^2^=0.165)  .062  (*P*=.292)  (f^2^=0.005)  [.356,.062]  .294  (*P*=.034) | .356  (*P*=.002)  (f^2^=0.165)  .267  (*P*=.026)  (f^2^=0.073)  [-.291,.295]  .089  (*P*=.324) | .356  (*P*=.002)  (f^2^=0.165)  .121  (*P*=.098)  (f^2^=0.020)  [-.221,.221]  .235  (*P*=.038) | .356  (*P*=.002)  (f^2^=0.165)  .213  (*P*=.012)  (f^2^=0.089)  [-.218,.227]  .143  (*P*=.146) | .356  (*P*=.002)  (f^2^=0.165)  .189  (*P*=.039)  (f^2^=0.047)  [-.241,.245]  .167  (*P*=.132) | .062  (*P*=.292)  (f^2^=0.005)  .267  (*P*=.026)  (f^2^=0.073)  [-.278,.284]  -.205  (*P*=.118) | .062  (*P*=.292)  (f^2^=0.005)  .121  (*P*=.098)  (f^2^=0.020)  [-.231,.225]  -.059  (*P*=.335) | .062  (*P*=.292)  (f^2^=0.005)  .213  (*P*=.012)  (f^2^=0.089)  [-.235,.234]  -.151  (*P*=.143) | .062  (*P*=.292)  (f^2^=0.005)  .189  (*P*=.039)  (f^2^=0.047)  [-.250,.253]  -.127  (*P*=.210) | .267  (*P*=.026)  (f^2^=0.073)  .121  (*P*=.098)  (f^2^=0.020)  [-.263,.251]  .146  (*P*=.183) | .267  (*P*=.026)  (f^2^=0.073)  .213  (*P*=.012)  (f^2^=0.089)  [-.258,.247]  .054  (*P*=.367) | .267  (*P*=.026)  (f^2^=0.073)  .189  (*P*=.039)  (f^2^=0.047)  [-.269,.275]  .078  (*P*=.308) | .121  (*P*=.098)  (f^2^=0.020)  .213  (*P*=.012)  (f^2^=0.089)  [-.220,.228]  -.092  (*P*=.207) | .121  (*P*=.098)  (f^2^=0.020)  .189  (*P*=.039)  (f^2^=0.047)  [-.229,.234]  -.068  (*P*=.307) | .213  (*P*=.012)  (f^2^=0.089)  .189  (*P*=.039)  (f^2^=0.047)  [-.246,.253]  .024  (*P*=.427) | not assigned | not assigned | not assigned |
| H9b | -.016  (*P*=.455)  (f^2^=0.000)  -.249  (*P*=.031)  (f^2^=0.048)  [-.296,.300]  .233  (*P*=.107) | -.016  (*P*=.455)  (f^2^=0.000)  -.495  (*P*<.001)  (f^2^=0.216)  [-.320,.316]  .480  (*P*=.006) | -.016  (*P*=.455)  (f^2^=0.000)  -.325  (*P*<.001)  (f^2^=0.114)  [-.276,.256]  .310  (*P*=.022) | -.016  (*P*=.455)  (f^2^=0.000)  -.134  (*P*=.161)  (f^2^=-0.089)  [-.332,.326]  .119  (*P*=.278) | -.016  (*P*=.455)  (f^2^=0.000)  -.354  (*P*<.001)  (f^2^=0.149)  [-.294,.283]  .338  (*P*=.026) | -.249  (*P*=.031)  (f^2^=0.048)  -.495  (*P*<.001)  (f^2^=0.216)  [-.306,.301]  .246  (*P*=.091) | -.249  (*P*=.031)  (f^2^=0.048)  -.325  (*P*<.001)  (f^2^=0.114)  [-.257,.259]  .077  (*P*=.304) | -.249  (*P*=.031)  (f^2^=0.048)  -.134  (*P*=.161)  (f^2^=-0.089)  [-.320,.305]  -.114  (*P*=.273) | -.249  (*P*=.031)  (f^2^=0.048)  -.354  (*P*<.001)  (f^2^=0.149)  [.278,.279]  .105  (*P*=.271) | -.495  (*P*<.001)  (f^2^=0.216)  -.325  (*P*<.001)  (f^2^=0.114)  [-.238,.239]  -.170  (*P*=.127) | -.495  (*P*<.001)  (f^2^=0.216)  -.134  (*P*=.161)  (f^2^=-0.089)  [-.322,.328]  .361  (*P*=.033) | -.495  (*P*<.001)  (f^2^=0.216)  -.354  (*P*<.001)  (f^2^=0.149)  [-.268,.260]  -.141  (*P*=.195) | -.325  (*P*<.001)  (f^2^=0.114)  -.134  (*P*=.161)  (f^2^=-0.089)  [-.266,.259]  -.191  (*P*=.112) | -.325  (*P*<.001)  (f^2^=0.114)  -.354  (*P*<.001)  (f^2^=0.149)  [-.227,.234]  .029  (*P*=.428) | -.134  (*P*=.161)  (f^2^=-0.089)  -.354  (*P*<.001)  (f^2^=0.149)  [-.288,.283]  .220  (*P*=.099) | not assigned | not assigned | not assigned |
| H9c | .043  (*P*=.304)  (f^2^=0.005)  .390  (*P*<.001)  (f^2^=0.257)  [-.235,.238]  -.347  (*P*=.006) | .043  (*P*=.304)  (f^2^=0.005)  .172  (*P*=.039)  (f^2^=0.049)  [-.215,.213]  -.129  (*P*=.171) | .043  (*P*=.304)  (f^2^=0.005)  .146  (*P*=.031)  (f^2^=0.038)  [-.175,.184]  -.103  (*P*=.169) | .043  (*P*=.304)  (f^2^=0.005)  .139  (*P*=.055)  (f^2^=0.045)  [-.195,.198]  -.096  (*P*=.215) | .043  (*P*=.304)  (f^2^=0.005)  .265  (*P*<.001)  (f^2^=0.161)  [-.169,.168]  -.221  (*P*=.014) | .390  (*P*<.001)  (f^2^=0.257)  .172  (*P*=.039)  (f^2^=0.049)  [-.221,.219]  .218  (*P*=.052) | .390  (*P*<.001)  (f^2^=0.257)  .146  (*P*=.031)  (f^2^=0.038)  [-.207,.203]  .245  (*P*=.025) | .390  (*P*<.001)  (f^2^=0.257)  .139  (*P*=.055)  (f^2^=0.045)  [-.228,.222]  .251  (*P*=.032) | .390  (*P*<.001)  (f^2^=0.257)  .265  (*P*<.001)  (f^2^=0.161)  [-.195,.196]  .126  (*P*=.149) | .172  (*P*=.039)  (f^2^=0.049)  .146  (*P*=.031)  (f^2^=0.038)  [-.200,.200]  .026  (*P*=.429) | .172  (*P*=.039)  (f^2^=0.049)  .139  (*P*=.055)  (f^2^=0.045)  [-.206,.195]  .033  (*P*=.385) | .172  (*P*=.039)  (f^2^=0.049)  .265  (*P*<.001)  (f^2^=0.161)  [-.180,.182]  -.092  (*P*=.202) | .146  (*P*=.031)  (f^2^=0.038)  .139  (*P*=.055)  (f^2^=0.045)  [-.198,.194]  .007  (*P*=.473) | .146  (*P*=.031)  (f^2^=0.038)  .265  (*P*<.001)  (f^2^=0.161)  [-.164,.156]  -.119  (*P*=.118) | .139  (*P*=.055)  (f^2^=0.045)  .265  (*P*<.001)  (f^2^=0.161)  [-.172,.168]  -.125  (*P*=.116) | not assigned | not assigned | not assigned |
| H9d | -.315  (*P*<.001)  (f^2^=0.110)  -.200  (*P*=.034)  (f^2^=0.041)  [-.235,.245]  -.116  (*P*=.205) | -.315  (*P*<.001)  (f^2^=0.110)  -.233  (*P*=.018)  (f^2^=0.057)  [-.222,.218]  -.082  (*P*=.265) | -.315  (*P*<.001)  (f^2^=0.110)  -.218  (*P*=.022)  (f^2^=0.050)  [-.231,.218]  -.097  (*P*=.242) | -.315  (*P*<.001)  (f^2^=0.110)  -.132  (*P*=.167)  (f^2^=0.018)  [-.222,.215]  -.183  (*P*=.095) | -.315  (*P*<.001)  (f^2^=0.110)  -.162  (*P*=.098)  (f^2^=0.027)  [-.236,.240]  -.153  (*P*=.152) | -.200  (*P*=.034)  (f^2^=0.041)  -.233  (*P*=.018)  (f^2^=0.057)  [-.256,.258]  .034  (*P*=.419) | -.200  (*P*=.034)  (f^2^=0.041)  -.218  (*P*=.022)  (f^2^=0.050)  [-.257,.259]  .019  (*P*=.447) | -.200  (*P*=.034)  (f^2^=0.041)  -.132  (*P*=.167)  (f^2^=0.018)  [-.288,.289]  -.067  (*P*=.357) | -.200  (*P*=.034)  (f^2^=0.041)  -.162  (*P*=.098)  (f^2^=0.027)  [-.294,.281]  -.037  (*P*=.428) | -.233  (*P*=.018)  (f^2^=0.057)  -.218  (*P*=.022)  (f^2^=0.050)  [-.256,.243]  -.015  (*P*=.479) | -.233  (*P*=.018)  (f^2^=0.057)  -.132  (*P*=.167)  (f^2^=0.018)  [-.273,.283]  -.101  (*P*=.271) | -.233  (*P*=.018)  (f^2^=0.057)  -.162  (*P*=.098)  (f^2^=0.027)  [-.284,.290]  -.071  (*P*=.346) | -.218  (*P*=.022)  (f^2^=0.050)  -.132  (*P*=.167)  (f^2^=0.018)  [-.263,.264]  -.086  (*P*=.281) | -.218  (*P*=.022)  (f^2^=0.050)  -.162  (*P*=.098)  (f^2^=0.027)  [-.262,.273]  -.056  (*P*=.362) | -.132  (*P*=.167)  (f^2^=0.018)  -.162  (*P*=.098)  (f^2^=0.027)  [-.306,.300]  .030  (*P*=.432) | not assigned | not assigned | not assigned |
| H9e | -.046  (*P*=.291)  (f^2^=0.006)  .048  (*P*=.359)  (f^2^=0.003)  [-.271,.265]  -.094  (*P*=.287) | -.046  (*P*=.291)  (f^2^=0.006)  .206  (*P*=.018)  (f^2^=0.076)  [-.212,.207]  -.252  (*P*=.023) | -.046  (*P*=.291)  (f^2^=0.006)  .157  (*P*=.004)  (f^2^=0.066)  [-.193,.188]  -.203  (*P*=.044) | -.046  (*P*=.291)  (f^2^=0.006)  -.077  (*P*=.170)  (f^2^=0.022)  [-.184,.187]  .031  (*P*=.397) | -.046  (*P*=.291)  (f^2^=0.006)  .297  (*P*=.008)  (f^2^=0.130)  [-.238,.239]  -.343  (*P*=.005) | .048  (*P*=.359)  (f^2^=0.003)  .206  (*P*=.018)  (f^2^=0.076)  [-.240,.249]  -.158  (*P*=.141) | .048  (*P*=.359)  (f^2^=0.003)  .157  (*P*=.004)  (f^2^=0.066)  [-.199,.199]  -.109  (*P*=.186) | .048  (*P*=.359)  (f^2^=0.003)  -.077  (*P*=.170)  (f^2^=0.022)  [-.241,.250]  .125  (*P*=.198) | .048  (*P*=.359)  (f^2^=0.003)  .297  (*P*=.008)  (f^2^=0.130)  [-.298,.304]  -.249  (*P*=.087) | .206  (*P*=.018)  (f^2^=0.076)  .157  (*P*=.004)  (f^2^=0.066)  [-.160,.161]  .050  (*P*=.298) | .206  (*P*=.018)  (f^2^=0.076)  -.077  (*P*=.170)  (f^2^=0.022)  [-.218,.218]  .283  (*P*=.109) | .206  (*P*=.018)  (f^2^=0.076)  .297  (*P*=.008)  (f^2^=0.130)  [-.269,.264]  -.091  (*P*=.307) | .157  (*P*=.004)  (f^2^=0.066)  -.077  (*P*=.170)  (f^2^=0.022)  [-.169,.167]  .234  (*P*=.009) | .157  (*P*=.004)  (f^2^=0.066)  .297  (*P*=.008)  (f^2^=0.130)  [-.198,.194]  -.140  (*P*=.126) | -.077  (*P*=.170)  (f^2^=0.022)  .297  (*P*=.008)  (f^2^=0.130)  [-.252,.265]  -.374  (*P*=.005) | not assigned | not assigned | not assigned |
| H10a | -.183  (*P*=.156)  (f^2^=0.047)  -.461  (*P*<.001)  (f^2^=0.354)  [-.286,.281]  .278  (*P*=.052) | -.183  (*P*=.156)  (f^2^=0.047)  -.459  (*P*<.001)  (f^2^=0.290)  [-.283,.280]  .276  (*P*=.052) | -.183  (*P*=.156)  (f^2^=0.047)  -.308  (*P*<.001)  (f^2^=0.138)  [-.274,.284]  .125  (*P*=.241) | -.183  (*P*=.156)  (f^2^=0.047)  -.448  (*P*<.001)  (f^2^=0.427)  [-.288,.298]  .266  (*P*=.068) | -.183  (*P*=.156)  (f^2^=0.047)  -.446  (*P*<.001)  (f^2^=0.271)  [-.288,.289]  .263  (*P*=.067) | -.461  (*P*<.001)  (f^2^=0.354)  -.459  (*P*<.001)  (f^2^=0.290)  [-.204,.211]  -.002  (*P*=.480) | -.461  (*P*<.001)  (f^2^=0.354)  -.308  (*P*<.001)  (f^2^=0.138)  [-.174,.169]  -.154  (*P*=.079) | -.461  (*P*<.001)  (f^2^=0.354)  -.448  (*P*<.001)  (f^2^=0.427)  [-.198,.202]  -.013  (*P*=.449) | -.461  (*P*<.001)  (f^2^=0.354)  -.446  (*P*<.001)  (f^2^=0.271)  [-.200,.200]  -.015  (*P*=.451) | -.459  (*P*<.001)  (f^2^=0.290)  -.308  (*P*<.001)  (f^2^=0.138)  [-.216,.204]  -.151  (*P*=.133) | -.459  (*P*<.001)  (f^2^=0.290)  -.448  (*P*<.001)  (f^2^=0.427)  [-.216,.226]  -.011  (*P*=.471) | -.459  (*P*<.001)  (f^2^=0.290)  -.446  (*P*<.001)  (f^2^=0.271)  [-.227,.219]  -.013  (*P*=.462) | -.308  (*P*<.001)  (f^2^=0.138)  -.448  (*P*<.001)  (f^2^=0.427)  [-.190,.197]  .141  (*P*=.118) | -.308  (*P*<.001)  (f^2^=0.138)  -.446  (*P*<.001)  (f^2^=0.271)  [-.192,.207]  .138  (*P*=.132) | -.448  (*P*<.001)  (f^2^=0.427)  -.446  (*P*<.001)  (f^2^=0.271)  [-.216,.217]  -.002  (*P*=.492) | -.103  (*P*=.190)  (f^2^=0.013)  -.158  (*P*=.046)  (f^2^=0.057)  [-.292,.281]  .055  (*P*=.377) | -.103  (*P*=.190)  (f^2^=0.013)  -.265  (*P*=.016)  (f^2^=0.089)  [-.301,.284]  .161  (*P*=.177) | -.158  (*P*=.046)  (f^2^=0.057)  -.265  (*P*=.016)  (f^2^=0.089)  [-.232,.239]  .106  (*P*=.237) |
| H10b | -.075  (*P*=.224)  (f^2^=0.013)  -.015  (*P*=.443)  (f^2^=0.000)  [-.215,.221]  -.060  (*P*=.334) | -.075  (*P*=.224)  (f^2^=0.013)  -.082  (*P*=.157)  (f^2^=0.013)  [-.164,.160]  .007  (*P*=.462) | -.075  (*P*=.224)  (f^2^=0.013)  -.040  (*P*=.288)  (f^2^=0.003)  [-.180,.178]  -.035  (*P*=.382) | -.075  (*P*=.224)  (0f^2^=0.013)  -.056  (*P*=.288)  (f^2^=0.005)  [-.178,.176]  -.019  (*P*=.425) | -.075  (*P*=.224)  (f^2^=0.013)  .018  (*P*=.416)  (f^2^=0.001)  [-.196,.195]  -.093  (*P*=.225) | -.015  (*P*=.443)  (f^2^=0.000)  -.082  (*P*=.157)  (f^2^=0.013)  [-.206,.199]  .067  (*P*=.331) | -.015  (*P*=.443)  (f^2^=0.000)  -.040  (*P*=.288)  (f^2^=0.003)  [-.211,.219]  .025  (*P*=.427) | -.015  (*P*=.443)  (f^2^=0.000)  -.056  (*P*=.288)  (f^2^=0.005)  [-.232,.234]  .041  (*P*=.380) | -.015  (*P*=.443)  (f^2^=0.000)  .018  (*P*=.416)  (f^2^=0.001)  [-.233,.228]  -.033  (*P*=.410) | -.082  (*P*=.157)  (f^2^=0.013)  -.040  (*P*=.288)  (f^2^=0.003)  [-.161,.157]  -.042  (*P*=.340) | -.082  (*P*=.157)  (f^2^=0.013)  -.056  (*P*=.288)  (f^2^=0.005)  [-.198,.201]  -.027  (*P*=.415) | -.082  (*P*=.157)  (f^2^=0.013)  .018  (*P*=.416)  (f^2^=0.001)  [-.198,.193]  -.100  (*P*=.210) | -.040  (*P*=.288)  (f^2^=0.003)  -.056  (*P*=.288)  (f^2^=0.005)  [-.175,.179]  .015  (*P*=.440) | -.040  (*P*=.288)  (f^2^=0.003)  .018  (*P*=.416)  (f^2^=0.001)  [-.194,.197]  -.058  (*P*=.004) | -.056  (*P*=.288)  (f^2^=0.005)  .018  (*P*=.416)  (f^2^=0.001)  [-.215,.213]  -.074  (*P*=.284) | -.228  (*P*=.011)  (f^2^=0.077)  -.068  (*P*=.289)  (f^2^=0.007)  [-.259,.266]  -.159  (*P*=.163) | -.228  (*P*=.011)  (f^2^=0.077)  .078  (*P*=.352)  (f^2^=0.005)  [-.383,.360]  -.306  (*P*=.096) | -.068  (*P*=.289)  (f^2^=0.007)  .078  (*P*=.352)  (f^2^=0.005)  [-.386,.384]  -.147  (*P*=.278) |
| H10c | -.002  (*P*=.492)  (f^2^=0.000)  -.006  (*P*=.475)  (f^2^=0.000)  [-.206,.206]  .004  (*P*=.489) | -.002  (*P*=.492)  (f^2^=0.000)  .139  (*P*=.069)  (f^2^=0.042)  [-.167,.162]  -.141  (*P*=.086) | -.002  (*P*=.492)  (f^2^=0.000)  -.079  (*P*=.082)  (f^2^=0.016)  [-.181,.191]  .077  (*P*=.260) | -.002  (*P*=.492)  (f^2^=0.000)  .057  (*P*=.197)  (f^2^=0.010)  [-.169,.162]  -.059  (*P*=.279) | -.002  (*P*=.492)  (f^2^=0.000)  -.017  (*P*=.425)  (f^2^=0.001)  [-.173,.180]  .015  (*P*=.446) | -.006  (*P*=.475)  (f^2^=0.000)  .139  (*P*=.069)  (f^2^=0.042)  [-.217,.208]  -.145  (*P*=.139) | -.006  (*P*=.475)  (f^2^=0.000)  -.079  (*P*=.082)  (f^2^=0.016)  [-.178,.171]  .073  (*P*=.251) | -.006  (*P*=.475)  (f^2^=0.000)  .057  (*P*=.197)  (f^2^=0.010)  [-.179,.175]  -.063  (*P*=.295) | -.006  (*P*=.475)  (f^2^=0.000)  -.017  (*P*=.425)  (f^2^=0.001)  [-.203,.204]  .011  (*P*=.466) | .139  (*P*=.069)  (f^2^=0.042)  -.079  (*P*=.082)  (f^2^=0.016)  [-.216,.204]  .218  (*P*=.014) | .139  (*P*=.069)  (f^2^=0.042)  .057  (*P*=.197)  (f^2^=0.010)  [-.193,.185]  .082  (*P*=.239) | .139  (*P*=.069)  (f^2^=0.042)  -.017  (*P*=.425)  (f^2^=0.001)  [-.194,.192]  .156  (*P*=.087) | -.079  (*P*=.082)  (f^2^=0.016)  .057  (*P*=.197)  (f^2^=0.010)  [-.149,.151]  -.136  (*P*=.069) | -.079  (*P*=.082)  (f^2^=0.016)  -.017  (*P*=.425)  (f^2^=0.001)  [-.158,.160]  -.062  (*P*=.272) | .057  (*P*=.197)  (f^2^=0.010)  -.017  (*P*=.425)  (f^2^=0.001)  [-.182,.183]  .074  (*P*=.251) | -.250  (*P*=.026)  (f^2^=0.073)  .001  (*P*=.497)  (f^2^=0.000)  [-.317,.300]  .251  (*P*=.092) | -.250  (*P*=.026)  (f^2^=0.073)  .275  (*P*=.025)  (f^2^=0.077)  [-.365,.374]  -.524  (*P*=.007) | .001  (*P*=.497)  (f^2^=0.000)  .275  (*P*=.025)  (f^2^=0.077)  [-.329,.337]  -.274  (*P*=.089) |
