# Supplementary figures and images for: Acceptance of AI in Health Care for Short- and Long-Term Treatments: Pilot Development Study of an Integrated Theoretical Model
Source: JMIR Form Res. 2024 Jul 18;8:e48600. doi: 10.2196/48600 (PMC11294784; doi:10.2196/48600)

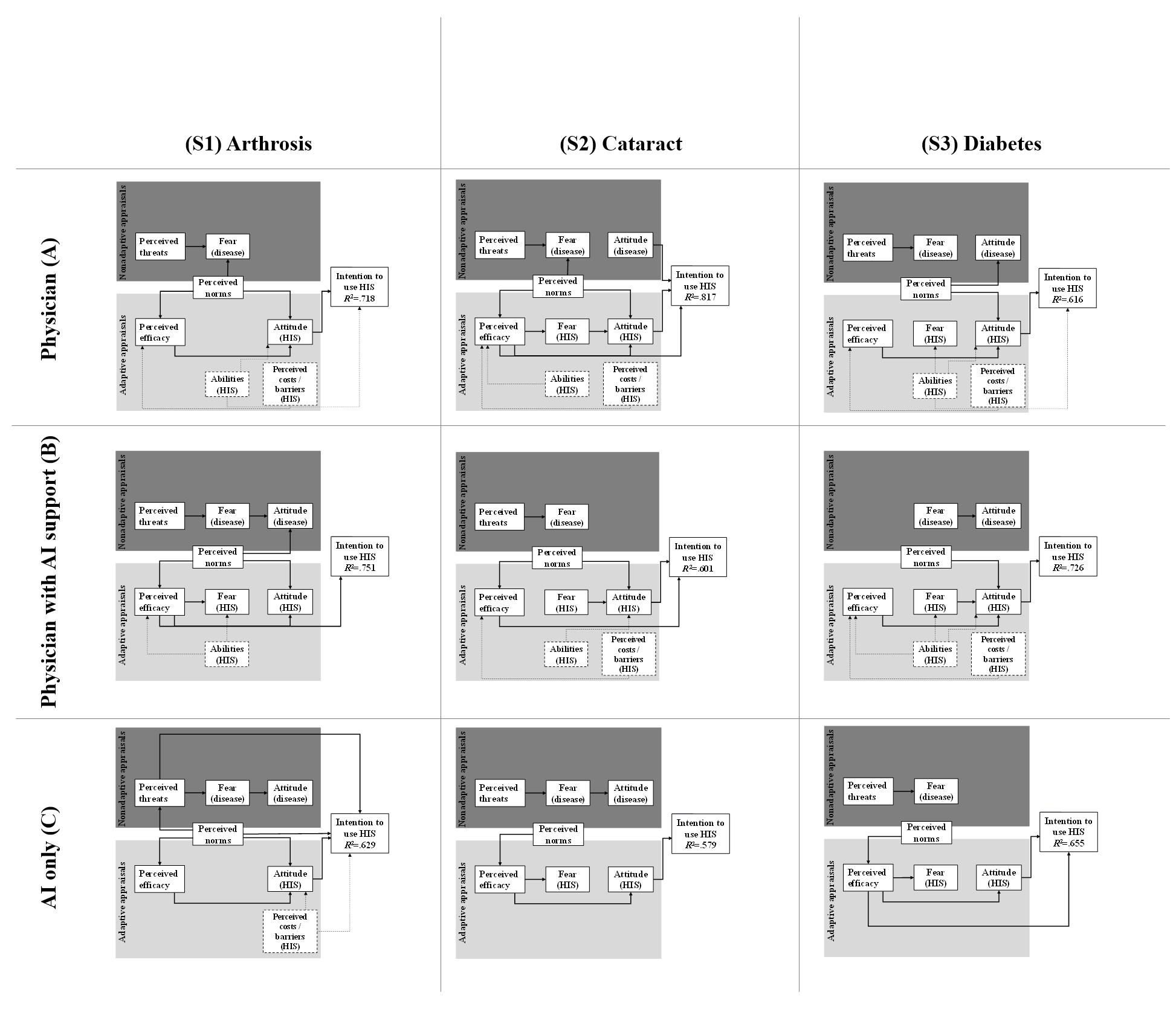

Supplement: Multimedia Appendix 2 [file formative_v8i1e48600_app2.png]
